# Low Plasma Citrate Levels and Specific Transcriptional Signatures Associated with Quiescence of CD34^+^ Progenitors Predict Azacitidine Therapy Failure in MDS/AML Patients

**DOI:** 10.3390/cancers13092161

**Published:** 2021-04-30

**Authors:** Pavla Koralkova, Monika Belickova, David Kundrat, Michaela Dostalova Merkerova, Zdenek Krejcik, Katarina Szikszai, Monika Kaisrlikova, Jitka Vesela, Pavla Vyhlidalova, Jan Stetka, Alzbeta Hlavackova, Jiri Suttnar, Patrik Flodr, Jan Stritesky, Anna Jonasova, Jaroslav Cermak, Vladimir Divoky

**Affiliations:** 1 Department of Biology, Faculty of Medicine and Dentistry, Palacky University Olomouc, 775 15 Olomouc, Czech Republic; pavla.koralkova@upol.cz (P.K.); vyhlidalova.pavla@gmail.com (P.V.); stetka.j@gmail.com (J.S.); 2 Institute of Hematology and Blood Transfusion, 128 20 Prague, Czech Republic; david.kundrat@uhkt.cz (D.K.); michaela.merkerova@uhkt.cz (M.D.M.); zdenek.krejcik@uhkt.cz (Z.K.); forgacova.katarina@gmail.com (K.S.); monika.hruba@uhkt.cz (M.K.); jitka.vesela@uhkt.cz (J.V.); alzbeta.hlavackova@uhkt.cz (A.H.); jiri.suttnar@uhkt.cz (J.S.); jaroslav.cermak@uhkt.cz (J.C.); 3 First Faculty of Medicine, Charles University, 121 08 Prague, Czech Republic; 4 Department of Clinical and Molecular Pathology, Faculty of Medicine and Dentistry, Palacky University Olomouc and University Hospital Olomouc, 775 15 Olomouc, Czech Republic; patrik.flodr@upol.cz; 5 Institute of Pathology, First Faculty of Medicine Charles University and General University Hospital, 128 08 Prague, Czech Republic; Jan.Stritesky@vfn.cz; 6 First Department of Medicine, First Faculty of Medicine Charles University and General University Hospital, 128 08 Prague, Czech Republic; anna.jonasova@vfn.cz

**Keywords:** myelodysplastic syndromes, azacitidine therapy, metabolic signature, IDH2, histone acetylation

## Abstract

**Simple Summary:**

Epigenetic drugs, such as azacitidine (AZA), hold promise in the treatment of myelodysplastic syndromes (MDS) and acute myeloid leukemia (AML), however, the mechanisms predicting the patients’ response to AZA is not completely understood. Quiescence of hematopoietic CD34^+^ progenitors has been proposed as a predictive factor for AZA therapy failure in MDS/AML patients, but the interplay between CD34^+^ cell cycle status and their metabolic signature in a predisposition to AZA (non)responsiveness remains unclear. Our data on patients with MDS or AML with myelodysplasia-related changes (AML-MRC) suggest that AZA-responders have actively cycling CD34^+^ cells poised for erythro-myeloid differentiation, with high metabolic activity controlling histone acetylation. Conversely, the patients who progressed early on AZA therapy revealed quiescence signature of their CD34^+^ cells, with signs of reduced metabolically-controlled acetylation of histones needed for transcription-permissive chromatin configuration. Our study delineates plasma citrate levels and CD34^+^ cells’ transcriptional signatures associated with cycling status and metabolic characteristics as factors predicting the response to AZA monotherapy in MDS/AML-MRC patients.

**Abstract:**

To better understand the molecular basis of resistance to azacitidine (AZA) therapy in myelodysplastic syndromes (MDS) and acute myeloid leukemia with myelodysplasia-related changes (AML-MRC), we performed RNA sequencing on pre-treatment CD34^+^ hematopoietic stem/progenitor cells (HSPCs) isolated from 25 MDS/AML-MRC patients of the discovery cohort (10 AZA responders (RD), six stable disease, nine progressive disease (PD) during AZA therapy) and from eight controls. Eleven MDS/AML-MRC samples were also available for analysis of selected metabolites, along with 17 additional samples from an independent validation cohort. Except for two patients, the others did not carry isocitrate dehydrogenase (IDH)1/2 mutations. Transcriptional landscapes of the patients’ HSPCs were comparable to those published previously, including decreased signatures of active cell cycling and DNA damage response in PD compared to RD and controls. In addition, PD-derived HSPCs revealed repressed markers of the tricarboxylic acid cycle, with *IDH2* among the top 50 downregulated genes in PD compared to RD. Decreased citrate plasma levels, downregulated expression of the (ATP)-citrate lyase and other transcriptional/metabolic networks indicate metabolism-driven histone modifications in PD HSPCs. Observed histone deacetylation is consistent with transcription-nonpermissive chromatin configuration and quiescence of PD HSPCs. This study highlights the complexity of the molecular network underlying response/resistance to hypomethylating agents.

## 1. Introduction

Hypomethylating agents (HMAs) like azacitidine (AZA) and decitabine have become a standard treatment option for high-risk myelodysplastic syndromes (MDS) or acute myeloid leukemia (AML) patients (including AML with myelodysplasia-related changes (AML-MRC)) [1,2,3,4]. However, although multiple clinical trials with HMAs showed significant clinical and survival benefits, the overall outcome and long-term survival of these patients remain poor. Only ∼50% of HMAs-treated patients respond to the treatment and most responders eventually relapse [5,6,7]. A great effort has been made to identify clinical, cytogenetic or molecular markers for HMA response and prognosis prediction in patients receiving HMA therapy [8,9,10]. Assessment of epigenetic mutations and detailed DNA methylation patterns in MDS patients has recently enabled the identification of clinically relevant subtypes of MDS, as well as the selection of those patients who have an increased likelihood of sensitivity or resistance to HMA therapy [11,12,13,14]. However, association between these molecular factors and eventual response to HMAs is still incompletely understood and, in some cases, remains controversial [15,16,17,18]. It seems that treatment strategies with HMAs should consider differences in HMA cell metabolism within individual patients’ groups ([19,20]) and may include effective combinations of HMAs with other new drugs with activity against MDS/AML ([21,22,23,24,25,26]; recent clinical trials are reviewed in [27,28]).

We previously reported increased expression of multiple ribosomal genes in MDS patients with poor prognosis and resistance to AZA therapy in a different patient cohort [29]. Herein, we confirm these findings, as well as the findings of several other laboratories reporting on the molecular markers predicting (non)responsiveness to AZA therapy in MDS/AML [30,31,32]. In addition, we extend previous observations and propose that expression of specific enzymes involved in key cellular metabolic pathways, which maintain the activity of the epigenetic program important for differentiation potential of hematopoietic stem/progenitor cells (HSPCs) and transcription-permissive chromatin configuration, represent important factors predicting the sensitivity or resistance to AZA.

## 2. Patients

The discovery cohort included 25 MDS/AML-MRC patients. Their bone marrow (BM) specimens (*n* = 25) and peripheral blood plasma samples (*n* = 11) were obtained during routine clinical assessments. The validation cohort for plasma metabolites verification consisted of 17 patients with MDS/AML-MRC. All samples were collected before the first administration of AZA with a median of 20 days (see Appendix A for details). AZA was given at 75 mg/m^2^ subcutaneously for 7 days and repeated every 4 weeks. Patients in both discovery and validation cohorts were divided into three groups: patients with complete response, partial response or hematological improvement were considered as responders (“RD”), patients with stable disease as (“SD”), and the third group involved patients with disease progression (“PD”). The pooling of patients who achieved any kind of response into the RD group was performed on the basis of established procedures for assessing AZA responses [3,5]; supported by unsupervised hierarchical clustering analysis in patients’ discovery cohort and normal controls, based on differentially expressed genes (DEGs) (Figure S1). Detailed characteristics of patients’ cohorts are available in Tables S1–S3 and in Figures S2 and S3. All subjects provided informed consent, and the local ethics committee approved the study. As controls (CTRL), CD34^+^ cells from 8 healthy individuals were used for RNA sequencing (RNA-seq) and plasma samples from 12 age-matched healthy individuals were collected for targeted metabolic analyses. For other information see Appendix A and Supplementary Materials. Overall survival (OS) and progression-free survival (PFS) for individual patients’ groups are depicted in Figure S4.

## 3. Results

### 3.1. Gene Expression Signatures Suggest Non-Cycling Status and Diminished Differentiation of CD34^+^ HSPCs in Patients with AZA Treatment Failure

**Figure 1:**
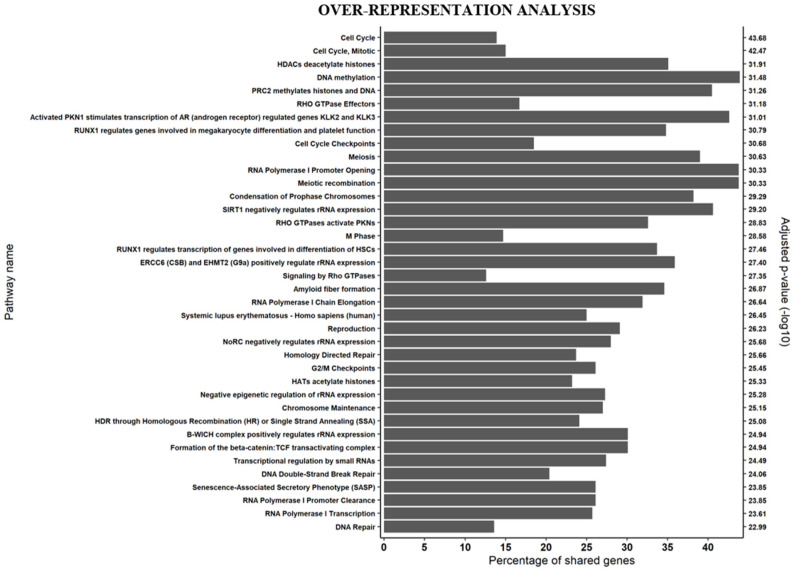
RNA-seq analysis on AZA pre-treatment MDS/AML-MRC CD34^+^ hematopoietic stem/progenitor cells isolated from 10 AZA-responders (RD) and nine patients with disease progression (PD). Identification of overrepresented biological processes in the differentially expressed genes (DEGs) between RD and PD (FDR < 0.05) compared to listed biological pathways using ConsensusPathDB with minimum overlap of 15 genes in pathway and *p*-value cut-off of 0.01. The percentage of DEGs associated with each biological process is shown along the *x*-axis and −log10-transformed *p*-values on the *y*-axis. To show the most significant results of over-representation analysis, a subset of full plot was constructed in which only pathways with a log transformed *p*-value of 23 and greater were kept. For the full plot see Figure S5a.

We first compared the biological function of DEGs between RD vs. PD, RD vs. CTRL and PD vs. CTRL, using over-representation analysis of DEGs with the ConsensusPathDB software [33]. These analyses covered a broad range of biological processes, with up to 84 processes in the RD vs. PD, 38 processes in the RD vs. CTRL and 122 processes in the PD vs. CTRL comparisons (Figure S5a–c). To show the most significantly affected networks characterizing differences between RD and PD, a subset of the full plot was constructed, in which only pathways with a log transformed *p*-value of 23 and greater were kept (Figure 1). Of the 38 biological processes, most of them were associated with cell cycle progression and related processes such as: chromatin condensation (nine), replication and DNA repair (five), epigenetic modification of chromatin and transcription (seven) and rRNA expression (four).

**Figure 2:**
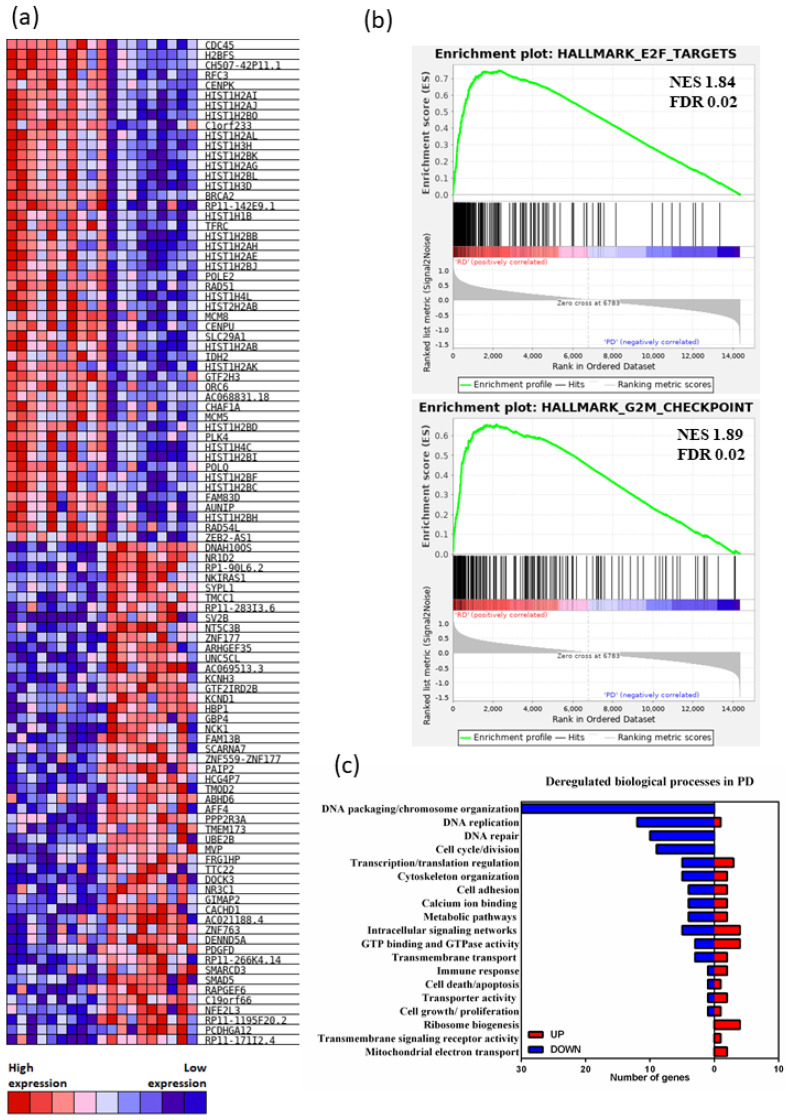
(**a**) Heatmap showing the top 50 up and downregulated genes in AZA-responders (RD) and patients with disease progression (PD), ranked by gene set enrichment analysis (GSEA). (**b**) GSEA indicating selected gene sets enriched in RD vs. PD patients’ groups (the top GSEA: Hallmark E2F targets gene set (MSigDB ref: M5925), the bottom plot: Hallmark G2/M checkpoint gene set (MSigDB ref: M5901)). The normalized enrichment scores (NES) and associated FDR values are depicted within the individual plots. (**c**) Functional annotation of 85 downregulated (blue) and 58 upregulated (red) differentially expressed genes in PD vs. RD patients’ groups (FDR < 0.02 cut-off).

The top 50 up and downregulated genes demonstrated predominant differential downregulation of expression of the replication-dependent histone *H2A* and *H2B* genes (found in histone gene cluster 1 (HIST1)) in PD patients’ HSPCs (Figure 2a); *H2A* and *H2B* gene isoform downregulation was also detected in the PD vs. CTRL gene set comparison (Figure S6).

The gene expression profile of RD CD34^+^ HSPCs strongly correlated with cell cycle related genes. GSEA analysis revealed that the RD CD34^+^ HSPCs are highly enriched for gene signatures of actively cycling cells in contrast to the PD CD34^+^ HSPCs (Figure 2b). Analysis of biological processes revealed that cell cycle genes as well as related processes, such as DNA replication and cell division, were strongly under-represented in PD CD34^+^ HSPCs (Figure 2c). Consistent with previous studies, these data suggested that CD34^+^ HSPCs populations of PD were mostly quiescent in their phenotype [31,34].

**Figure 3:**
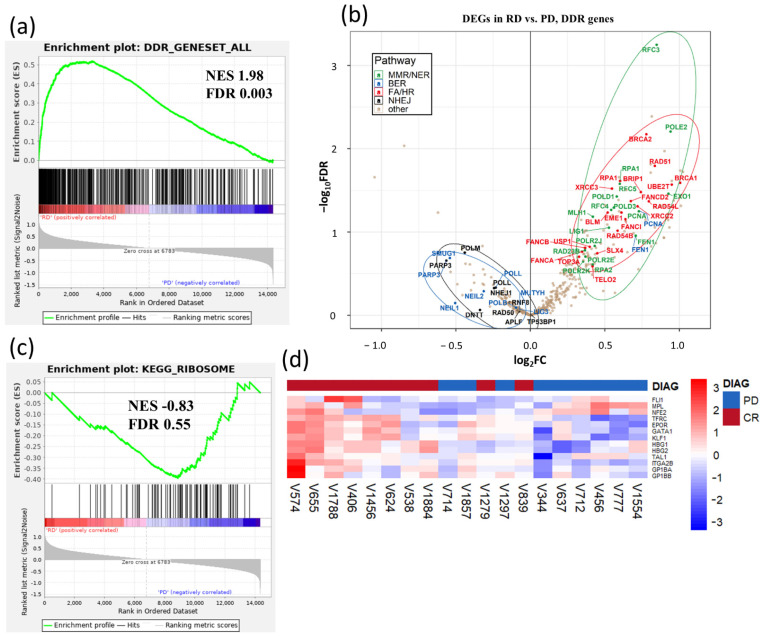
(**a**,**c**) Gene set enrichment analysis (GSEA) indicating selected gene sets enriched in CD34^+^ hematopoietic stem/progenitor cells (HSPCs) from AZA-responders (RD) and patients with progressive disease (PD). The top GSEA plot analyzes a set of genes implicated in DNA damage response (DDR) and repair pathways [36]. The bottom GSEA plot shows KEGG Ribosome gene set (MSigDB ref: M189)). The normalized enrichment scores (NES) and associated FDR values are depicted within the individual plots. (**b**) Volcano plot of DDR gene expression. The plot shows differential expression status of 276 DDR genes in HSPCs samples from RD vs. PD patients (for the associated heatmaps see Figure S7a,b). The most upregulated genes in the RD (i.e., downregulated in the PD) are towards the right; the most downregulated genes in the RD (i.e., upregulated in the PD) are towards the left. The subgroups of mismatch repair/nucleotide excision repair (MMR/NER), base excision repair (BER), Fanconi anemia and homologous recombination (FA/HR) and nonhomologous end-joining (NHEJ) gene clusters are labeled and circled in green/blue/red and black, respectively. The designation of genes into the subgroups is based on Pearl et al. [36]. (**d**) Hierarchical clustering heatmap of gene expression of 13 selected megakaryocytic/erythroid genes in AZA pre-treatment CD34^+^ HSPCs from RD and PD patients. The top bar indicates the RD patients in red and the PD patients in blue.

Quiescent, non-cycling hematopoietic stem cells are characterized by broadly attenuated multiple DNA damage response (DDR) and repair pathways [35]. To address possible impaired DDR in HSPCs of PD AZA non-responders, we analyzed the overall degree of DDR activation in HSPCs using transcriptional profiling of 276 DDR-related genes [36]. Heatmap representation (Figure S7a,b) and GSEA (Figure 3a) revealed an activated DDR signature in AZA responders, but largely a suppressed DDR signature in PD AZA patients. The overall DDR gene expression in SD patients was somewhere in between the RD and PD transcriptomes (Figure S7a,b).

To examine whether observed differences in the cycling/non-cycling characteristics of CD34^+^ HSPCs populations of RD vs. PD is reflected in the mechanisms of DNA repair activated in these cells, we evaluated transcripts of DNA repair genes distributed to categories as described [36]. DNA double strand repair genes represented the most differentially expressed gene set, as RD were enriched for homologous recombination (HR) and Fanconi anemia (FA) upregulated genes while PD activated nonhomologous end-joining (NHEJ) repair mechanism (Figure 3b). In fact, two members of HR genes, *RAD51* and *BRCA2*, were among the top 50 downregulated DEGs in PD AZA non-responders (Figure 2a). DNA single strand repair genes categories were differentially expressed as well: we observed significant enrichment for mismatch repair/nucleotide excision repair (MMR/NER) upregulated genes in the RD HSPCs and for base excision repair (BER) in the PD patients’ cells (Figure 3b). These data are consistent with the proliferative phenotype signature of RD HSPCs, as proliferating HSCs primarily employ FA/HR and MMR/NER, and quiescent phenotype signature of PD HSPCs, as quiescent HSCs primarily employ NHEJ and BER for DNA repair [37,38,39].

The most upregulated gene signature in the PD vs. RD patients (i.e., differentially downregulated in RD, although not reaching significant FDR value threshold) was the KEGG ribosome gene set (Figure 3c). This results corresponds to our earlier data [29]. Stevens et al. confirmed significant upregulation of ribosome signaling signature in malignant HSCs in advanced MDS [40]. The 16S ribosomal RNA gene, *MT-RNR2*, encoding anti-apoptotic peptide humanin [41], was the most upregulated DEG in the PD vs. RD comparison (Table S5). High expression of ribosomal genes was previously associated with HSC aging, with differentially overexpressed ribosomal proteins and RNA genes, and hypomethylation of rRNA genes in aged HSCs [42]. There are also other similarities between PD cells with a quiescent signature and aged HSCs. This includes the differential downregulation of genes implicated in DNA repair, chromosome organization, DNA replication and cell cycle (Figure 2c) in PD cells which resembles aged HSCs where such a transcriptome reflects the diminished differentiation capacity of these cells [42].

In this regard, the genes of the megakaryocytic/erythroid program [43] were expressed preferentially in RD (Figure 3d). Among them, *HBG1* and *HBG2* had the highest average fold-change (FC) when the genes were ranked using logFC (Table S5), and were previously implicated in better AZA treatment outcome in MDS/AML patients [30]. Together, these data are consistent with impaired differentiation program in HSPCs of PD patients, whereas RD HSPCs are primed for myeloid differentiation.

### 3.2. Metabolic Signature Differences between AZA Pre-Treatment RD and PD Patients

Earlier studies investigated pathways linked to DNA methylation [16,18,21,44,45] and histone modification [46,47] affecting AZA responsiveness. As shown in Figure 1, processes related to epigenetic modifications of DNA and histones were significantly over-represented in DEGs between RD vs. PD patients. Mutations in epigenetic regulators (such as *DNMT3A*, *TET2*, *EZH2*, *ASXL1*) were identified in several patients, but their distributions and frequencies were either comparable across all patients’ groups or our cohort was not large enough to assess the possible contribution of these mutations to AZA-responsiveness (Tables S1 and S2), as published earlier [9,10,11,12,15,48,49].

**Figure 4:**
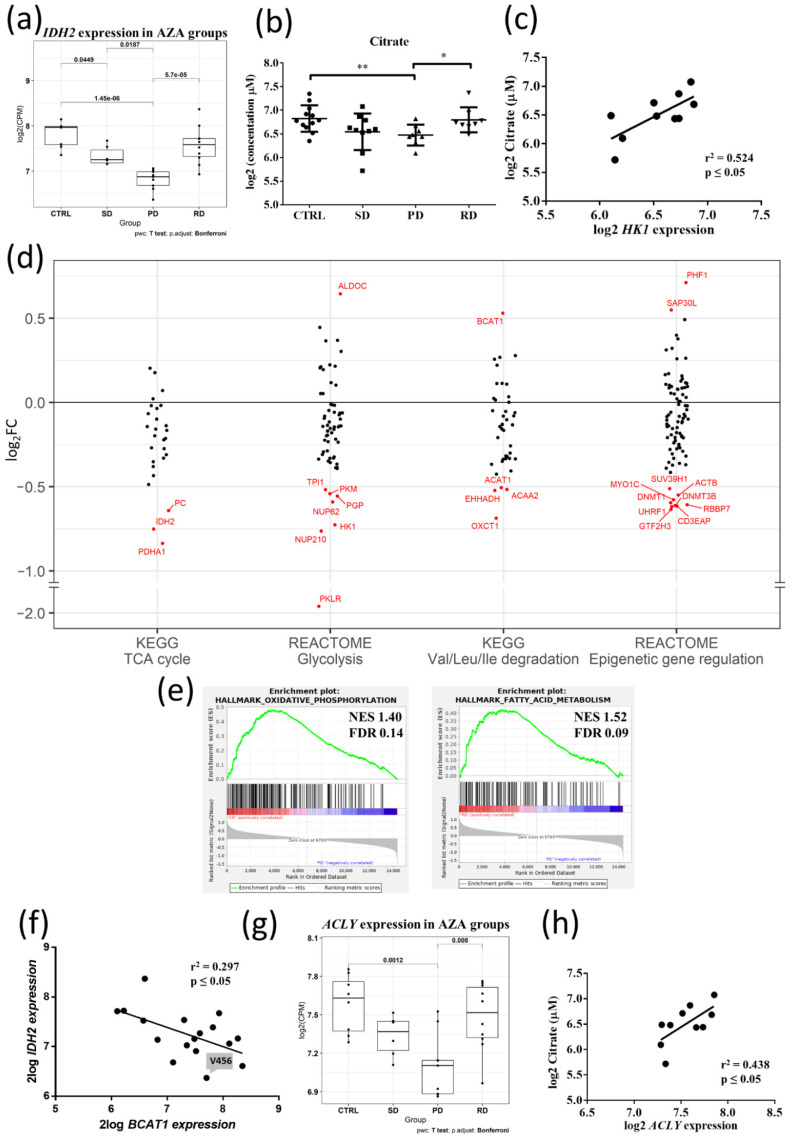
Characterization of metabolic signature of AZA pre-treatment MDS/AML-MRC patients. (**a**) Boxplot showing *IDH2* expression in CD34^+^ hematopoietic stem/progenitor cells (HSPCs) from patients of discovery cohort and controls. The box is defined by lower and upper quartile, and the line across the box represents median. Student’s *t*-test with Bonferroni correction. Statistical significance between individual patients’ groups (SD—stable disease, PD—progressive disease, RD—AZA-responders) and controls (CTRL) is shown in the graph. (**b**) Plasma citrate levels in AZA pre-treatment MDS/AML-MRC patients and controls. Patient samples from discovery (*n* = 10) and validation (*n* = 16) cohorts and controls (*n* = 12) were analyzed. Two patients with *IDH2* mutational status were excluded from statistical analysis. Citrate levels were significantly decreased in PD compared to RD patients and CTRL. Mann-Whitney U test, * *p* ≤ 0.05, ** *p* ≤ 0.01. (**c**) Correlation analysis between *HK1* expression and plasma citrate levels in AZA pre-treatment MDS/AML-MRC patients. Data from the discovery cohort (*n* = 10, a patient with *IDH2* mutation was excluded) were analyzed using Pearson correlation and Linear Regression model. R^2^ value reflects the goodness of fit of the regression model to the data; *p*-value indicates statistical significance. (**d**) RNA-seq analysis of metabolic gene expression alteration between PD and RD patients of discovery cohort. A column scatter plot was constructed by plotting the log2 fold change (log2FC) of differentially expressed genes (PD versus RD) that are involved in key metabolic processes, including tricarboxylic acid (TCA) cycle (KEGG—hsa00020), glycolysis (REACTOME—R-HSA-70171), valine-leucine-isoleucine degradation (KEGG—hsa00280) and epigenetic gene regulation (REACTOME—R-HSA-212165). A positive log2FC value represents up-regulation in PD and a negative value represents down-regulation. Red dots indicate the most deregulated genes within a given pathway. (**e**) Gene set enrichment analysis (GSEA) plot for Hallmark gene sets oxidative phosphorylation (**left**) and fatty acid metabolism (**right**) in CD34^+^ HSPCs from RD vs. PD patients. (**f**) Correlation analysis between *IDH2* and *BCAT1* expression in CD34^+^ HSPCs from AZA pre-treatment MDS/AML-MRC patients. A notable outlier, patient V456 depicted by an arrow, showed discordance in the level of *IDH2* expression detected by RNA-seq and protein level detected by immunohistochemistry staining. Data from discovery cohort were analyzed using Pearson correlation and Linear Regression model. R^2^ value is defined as in 4c; *p*-value indicates statistical significance. (**g**) Boxplot showing *ACLY* expression in CD34^+^ HSPCs from patients of discovery cohort and controls. The box is defined as described in 4a. Student’s *t*-test with Bonferroni correction. Statistical significance between individual patients’ groups (SD, PD, RD) and controls (CTRL) is shown in the graph. (**h**) Correlation analysis between *ACLY* expression and plasma citrate levels in AZA pre-treatment MDS/AML-MRC patients. Data from the discovery cohort (*n* = 10, a patient with *IDH2* mutation was excluded) were analyzed using Pearson correlation and Linear Regression model. R^2^ value is defined as in 4c; *p*-value indicates statistical significance.

In addition to genetic mutations, activity and expression levels of DNA and histone modifiers are regulated by the cell’s metabolic state [50]. In this regard, alterations in pyrimidine metabolism were described as leading to AZA-resistance [20], and disruption of the tricarboxylic acid (TCA) cycle resulting from combinatory action of venetoclax + AZA was proposed to be responsible for causing elimination of AML stem cells [51]. Given the significant downregulation of *IDH2* in PD patients from our discovery cohort (Figure 4a), *IDH2* belonged to the most suppressed energy-associated genes and was also listed among the top 50 downregulated genes in PD HSPCs (Figure 2a), we hypothesized that dysregulation of energy metabolism is strongly linked to non-responsiveness and disease progression during AZA therapy. We performed measurements of TCA and glycolytic metabolite concentrations in the plasma of AZA-pre-treatment patients and normal controls, 11 of the MDS/AML-MRC samples from a discovery cohort (3 RD, 4 SD, 4 PD), along with additional 17 samples from the independent validation cohort (5 RD, 7 SD, 5 PD) and 12 controls, to provide sufficient statistical power in individual patient groups (Table S6). The principal component analysis (PCA) plot showed that a targeted plasma metabolic profile clearly differentiated PD from RD patients and from the controls (Figure S8a–c); the patient with *IDH2* mutation was excluded from further metabolic comparison tests. Citrate levels were significantly downregulated in PD vs. RD patients (*p* ≤ 0.05), and vs. CTRL (*p* ≤ 0.01) (Figure 4b). Furthermore, in PD vs. CTRL, we observed significant decrease in alpha-ketoglutarate (αKG) and succinate levels, while the concentrations of L- and D-2HG, were not significantly different (Table S6). No significant differences were observed in the plasma levels of αKG or succinate between PD vs. RD patients (Table S6). The expression of several key glycolytic enzymes was decreased in PD vs. RD (Figure S9), including *HK1*, which moderately correlated with decreased citrate levels (*p* ≤ 0.05, r^2^ = 0.524) (Figure 4c). Further scatter analysis of DEGs involved in energy metabolism showed transcriptional downregulation in PD HSPCs’ genes for multiple rate-limiting enzymes of glycolytic pathway and TCA cycle, as well as genes for enzymes that utilize pyruvate and directly link glycolysis and TCA cycle (Figure 4d). Furthermore, genes involved in oxidative phosphorylation and fatty acid metabolism showed enriched expression in RD vs. PD HSPCs (Figure 4e). These data suggested attenuated/impaired mitochondrial respiration (and linked pyrimidine biosynthesis, Figure S10) and glycolysis in PD HSPCs, and are consistent with overall decreased energy metabolism in non-cycling HSPCs [52]. Next, we tested if transcriptome of branched-chain amino acid (BCAA) catabolism, another relevant energy source in HSPCs [53], is differentially dysregulated between PD vs. RD patients. The column scatter plot (Figure 4d; for the associated heatmap see Figure S11) revealed upregulated branched-chain aminotransferase 1 (*BCAT1*) in the PD vs. RD patients. *BCAT1* has been implicated in leukemia stem cell (LSC) metabolism as its overexpression decreased intracellular αKG levels and caused DNA hypermethylation through altered TET activity; AML with high levels of *BCAT1* displayed a DNA hypermethylation phenotype similar to cases carrying a mutant IDH [54,55]. These data suggested that decreased expression of *IDH2* (otherwise non-mutated in our patients except for the excluded exceptions) and increased *BCAT1* are interlinked in our patients’ cohort (exhibiting the significance level of *p* < 0.05, but a weak linear relationship r^2^ = 0.297, Figure 4f; Appendix B). As the acetylation status of histones is tightly regulated with energy metabolism through availability of acetyl-Co A [56,57], we examined the expression of the key acetyl-CoA producing enzymes. Indeed, a gene for (ATP)-citrate lyase (*ACLY*), a central metabolic enzyme generating acetyl-Co A for histone acetylation [57,58], showed a significant decrease of gene expression in PD vs. RD patients and CTRL (Figure 4g) and moderate correlation with citrate levels (*p* ≤ 0.05, r^2^ = 0.438) (Figure 4h); the expression of acetyl-Co A synthases (*ACSS1/2*) did not change in response to *ACLY* downregulation in PD patients (Figure S12).

Previous studies validated DNMT1, a maintenance DNA methyltransferase, as a pharmacologic target of AZA therapy [59,60]. The *DNMT1* gene was among the most downregulated genes of epigenetic modifying enzymes in PD HSPCs (Figure 4d). This contrasts with previous study where increased but not decreased expression of *DNMT1* was associated with AZA resistance [61]. However, this association appears to be applicable to replicating cells; as active DNA replication is required for incorporation of AZA into DNA, enabling the subsequent trapping and degradation of DNMT1 [60]. Therefore, in replicating cells, AZA would be predicted to be less effective under conditions where DNMT1 level is higher and more effective when DNMT1 level is lower, so that its depletion by AZA is sufficient to exceed the minimal threshold required for cellular response (i.e., DNA demethylation) [62]. On the other hand, *DNMT1* expression increases during S phase in dividing cells [63,64,65], and therefore the observed downregulation of *DNMT1* (as well as downregulation of other DNA methyltransferase, *DNMT3B,* Figure 4d) in our PD patient group is primarily a consequence of HSPC non-cycling status and reduced replication.

### 3.3. Relationship between Gene Expression Signatures and Immunohistochemistry (IHC) of IDH2 and Acetylated Histone H3 Lysine 9

**Figure 5:**
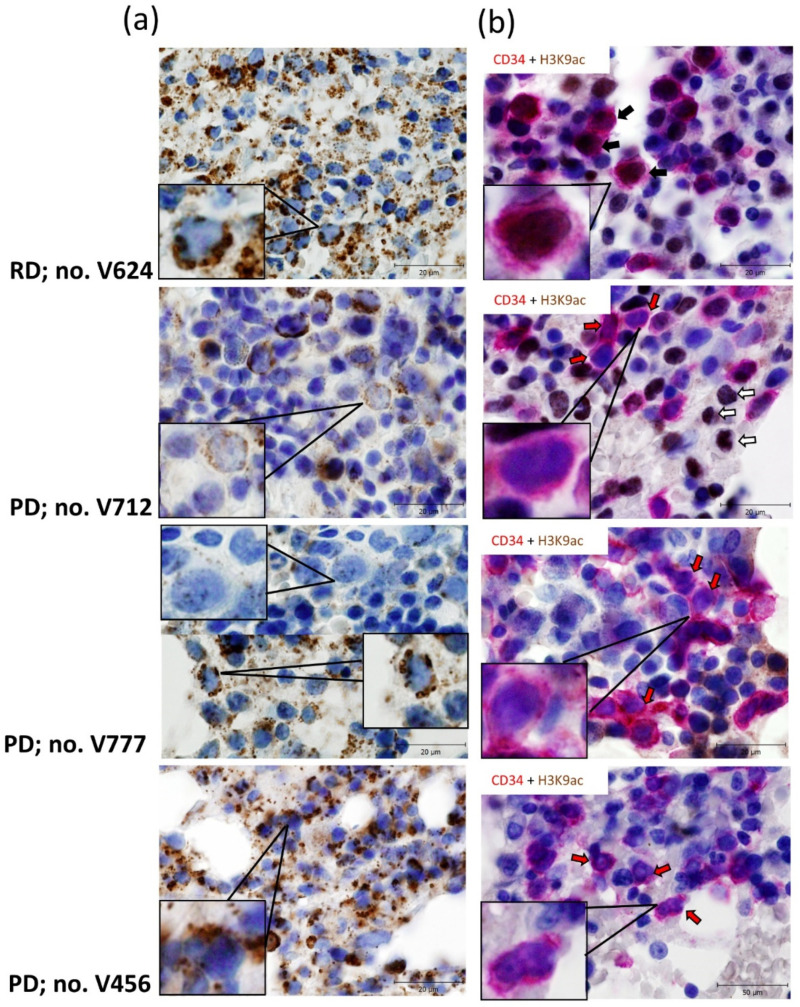
Representative IHC staining for IDH2 (**a**) and CD34 with histone H3 lysine 9 acetylation (H3K9ac) (**b**) in BM trephine biopsies of AZA pre-treatment MDS/AML-MRC patients. (**a**) Mitochondrial staining for IDH2 (brown) with hematoxylin nuclear counterstains (blue) in BM trephine biopsies from RD vs. PD patients. Upper panel: Strong mitochondrial staining for IDH2 in RD patient no. V624. Middle panels: Rare/weak IDH2 staining of PD patient no. V712; alternating clusters of either positively or negatively stained cells in PD patient no. V777. Lower panel: Intermediate/strong mitochondrial staining for IDH2 in PD patient no. V456. See Supplementary Table S1 for patients’ details. Scale bars, 20 μm. (**b**) Sequential double staining for CD34 (first primary antibody, visualized with the Envision FLEX HRP Magenta Substrate Chromogen System (red)) and H3K9ac (second primary antibody, visualized with DAB chromogen (brown)), with hematoxylin nuclear counterstains (blue). Individual sections show cells with specific membranous and diffuse cytoplasmic staining for CD34 [68] and nuclear H3K9ac staining. The arrows indicate examples of CD34 and H3K9ac double-positive cells (black arrows), CD34 positive cells (red arrows) and H3K9ac positive cells (white arrows). Upper panel: Co-expression of CD34 and H3K9ac in RD patient no. V624. Middle and lower panels: Distinct cells with immature nuclei stain for CD34 and with less immature nuclei for H3K9ac in three PD patients no. V712, V777, V456. See Supplementary Table S1 for patients’ details. Scale bars, 20 μm.

Finally, we examined whether decreased expression of *IDH2* and suppressed expression of the genes for enzymes positively regulating histone acetylation in the PD vs. RD patients’ cells are reflected in the protein regulation in vivo. BM biopsy paraffin sections of selected patients were stained for IDH2, IDH1, pan-acetyl lysine and acetylated histone H3 lysine 9 (H3K9ac), using antibodies specified in Table S4. First, IHC for mitochondrial marker, apoptosis-inducing factor (AIF, [66]), was used to evaluate the pattern of mitochondrial protein expression prior to staining with IDH2 antibody (data not shown). Next, we analyzed IDH2 and IDH1 protein expression in representative patients from our study cohorts. Our data confirmed correlation between *IDH2* expression detected by RNA-seq and protein level of IDH2 in a selected RD patient (Figure 5a). The pattern of IDH2 expression in selected PD samples revealed variable regional heterogeneity in examined BM sections. Of the three PD patients examined, the degree of correlation with transcriptomic analysis ranged from highly concordant (patient V712), moderately concordant (patient V777) to poor (patient V456, Figure 5a). Patient V456 demonstrated incongruity in IDH2 transcript/protein levels, despite low *IDH2* transcript his mitochondrial IDH2 staining revealed higher positivity, in line with aforementioned *IDH2/BCAT1* regulation depicted in Figure 4f. IDH1 expression does not seem to compensate for decreased IDH2 in the analyzed PD patients (Figure S13a).

Unlike IDH2, transcriptomic signatures and the expression of a specific histone acetylation marker were highly concordant. IHC staining of pan-acetyl lysine revealed brightly stained nuclei in the RD patient; in the PD patients’ biopsies, we observed barely detectable or rare pan-acetyl lysine nuclear immunoreactivity (Figure S13b). However, these IHC stains did not directly target histone acetylation and could not be directly related to CD34^+^ HSPC-based RNA-seq, because expression of CD34 within the MDS BM compartment is very variable and CD34^+^ HSPCs represent heterogeneous subpopulations of immature cells [40]. To further pinpoint which cells stained for histone acetylation in these biopsy sections are CD34^+^ cells, we used sequential double IHC staining for CD34 and H3K9ac, as the antigens of interest are located in different cellular compartments. We selected anti-H3K9 acetyl antibody for this experiment; H3K9ac is targeted by histone deacetylase SirT1 [67], and we observed significantly upregulated expression of *SIRT1* in PD vs. RD patients’ groups (Figure S14). As shown in Figure 5b, cells co-expressing CD34 and H3K9ac were detected only in analyzed RD patient; the sections of three PD patients clearly show CD34-positive cells (i.e., sporadic cells with immature nuclei) without H3K9ac staining, and vice versa, H3K9ac-positive cells (some cells with less immature nuclei) without expression of CD34. These data further support the relevance of our correlations between transcriptome signatures, metabolic activity and acetylation of histones in AZA pre-treatment MDS/AML-MRC patients.

## 4. Discussion

This study contributes to a better understanding of the mechanism by which metabolic properties associated with the quiescent state of malignant MDS/AML-MRC CD34^+^ progenitors affect AZA (non)responsiveness. Our data provide insights into the association between cell-cycle quiescence, cell metabolic rate and its link to epigenetic modifications, and pre-existing failure to respond to AZA treatment.

CD34^+^ malignant HSPCs in MDS as well as in AML exhibit largely quiescent phenotype [40,69,70]. Because these cells represent a highly heterogeneous pool of genetically and epigenetically abnormal stem cell subclones [71,72], the proportion of cycling/quiescent HSCs varies in individual patients [31,69]. In addition, the CD34^+^ compartment of BM cells in MDS/AML-MRC contains both the clonotypic (malignant) as well as non-malignant (normal) HSPCs [73], and the fact that we used unfractionated bulk of CD34^+^ cells for RNA-seq represents bias that could influence our results. However, this effect of sample bias can be estimated to be relatively low; the CD34^+^ compartment contained ≥97.5% of myeloid blasts (see Appendix C for details), which can be considered as mostly neoplastic, enriched for cytogenetically and functionally abnormal cells [71].

Recent studies suggested that the proportion of pre-AZA BM quiescent HSPCs represents a key predictor of treatment outcome in high risk MDS [31] and AML [34]. Other studies have documented altered cellular metabolism of quiescent HSCs (including malignant LSCs), mainly suppression of aerobic respiration (reviewed in [52,53]). As metabolic pathways are closely interconnected with cellular epigenetic modifications modulated by histone- and DNA-modifying enzymes [50,56,57], we sought to investigate the interplay between the cell cycle status and metabolic signature in predisposition to AZA therapy (non)responsiveness.

Using RNA-seq on pre-treatment CD34^+^ HSPCs isolated from 25 MDS/AML-MRC patients, we show that RD patients’ BM contains actively cycling cells poised for erythro-myeloid differentiation. In contrast, the PD patients’ CD34^+^ HSPCs display different properties: they reveal multiple gene expression signatures of a quiescent state. These gene expression characteristics include: repression of genes related to active cell cycling, replication and DDR. In this context, the suppression of cell cycle-dependent DNA repair pathways may render the PD patients’ stem cells vulnerable to the accumulation of DNA damage during the course of the disease [72] and aging [35] and thus represents a negative prognostic factor per se associated with the poor outcomes of these patients. In accordance with this concept, multiple properties of the PD patients’ cells resembled aged HSCs, including the most markedly differentially overexpressed ribosomal gene set [42]. These data are also consistent with the concept of premature aging of MDS HSCs [74] and progenitors [75]. The non-cycling status and low replication rate signature were also associated with decreased expression of the replication-dependent histone genes of the HIST1 cluster, which are the most represented genes among the PD patients’ top 50 downregulated genes. Their repression may be part of a gene network controlling quiescent state of CD34^+^ cells in this subset of patients [76].

We sought to determine the metabolic and epigenetic transcriptome characteristics of the PD HSPCs with a high quiescent population. We hypothesized that increased quiescence as an inherent feature of PD patients’ progenitors is associated with their decreased metabolic activity and impaired metabolism-driven modifications of histones and DNA needed for transcription-permissive chromatin configuration, which, in a feedforward loop, prevents the quiescent progenitors from cell cycle entry and differentiation. Our RNA-seq data revealed expression signatures of overall decreased energy metabolism in PD vs. RD patients. These signatures of suppressed metabolic pathways included aerobic respiration, glycolysis as well as fatty acid metabolism. On the other hand, the *BCAT1* gene, encoding enzyme catalyzing the amination of branched-chain keto acids [54], was differentially upregulated in PD patients, and its expression negatively correlated with the expression of *IDH2*, one of the top 50 downregulated genes in the PD vs. RD comparison. Both these enzymes are connected with αKG production, TET2-mediated DNA hydroxymethylation and other metabolism-driven modifications [53,55], suggesting that they contribute to the quiescence-associated epigenetic status of PD progenitors. Even though a previous study focused on one type of solid tumor did not find concordance between gene transcription and metabolite levels [77], another study on HSCs revealed strong positive correlation between transcriptome and proteome data sets, including metabolic enzymes and other components of metabolic pathways [43]. Indeed, plasma metabolic profiling, for which we acquired additional samples sourced from an independent MDS/AML-MRC validation cohort and controls, supported our transcriptome-based data. Using LC-MS/MS analysis, we observed altered plasma TCA cycle metabolite concentrations; decreased citrate (PD vs. RD and vs. CTRL) and αKG and succinate (PD vs. CTRL) in PD patients. IDH2 downregulation was consistent with decreased IDH2 protein detected by IHC on selected patients’ BM sections. The levels of 2-HG oncometabolite were not significantly different between the patients’ groups, which is in agreement with the non-mutant status of IDH1/2 in studied patients. It is known that hormonal differences between the sexes can alter the metabolic profile of blood plasma [78], and gender representations were not uniform in our cohorts. However, and importantly for our study, the metabolites discussed here are not significantly altered by hormonal differences between male and female patients [79,80], and therefore gender differences in patient groups likely did not affect the above plasma parameters.

Besides cell proliferation and DNA replication, epigenetic histone modifications were the most over-represented processes in the RD vs. PD comparison. Of the epigenetic modifications, histone lysine acetylation marks open chromatin and promotes active gene expression. These processes are dynamically regulated by histone modification enzymes, as well as availability of acetyl-CoA [81,82]. Glucose has been shown to be a major source for histone acetylation [58,81,82], via the pyruvate dehydrogenase complex (PDH) in the mitochondria, which converts pyruvate to acetyl-CoA. The *PDHA1* gene, encoding pyruvate dehydrogenase E1 subunit α 1, was, together with *IDH2*, the most differentially downregulated gene from the KEGG TCA cycle gene set in the PD vs. RD comparison. Acetyl-CoA in the form of citrate is oxidized via the TCA cycle, or alternatively it can be transported out of the mitochondria and diffuse into the nucleus, where it is cleaved by ACLY to reform acetyl-CoA, which is used to acetylate histones [57,58]. The *ACLY* expression was significantly downregulated in PD vs. RD patients and vs. CTRL. In line with these results, levels/expression of citrate and *ACLY*, the regulators of the histone acetylation state, moderately correlated. All these data collectively suggested metabolism-driven decreased histone acetylation, i.e., epigenetic modifications associated with transcription-nonpermissive chromatin configuration in PD HSPCs maintaining their quiescence.

Cross-talk between DNA methylation and hypoacetylated histones in maintaining gene silencing is well described [83]. Our antibody staining confirmed an overall decrease in nuclear histone acetylation in the assayed BM biopsies of PD patients. In striking contrast, the biopsy of an RD patient revealed high nuclear lysine acetylation, including H3K9 acetylation. These results together with the metabolic signature discussed above collectively indicate active regulation of metabolism-mediated histone acetylation in proliferating HSPCs in RD patients. Impact of decreased metabolic activity on histone acetylation in BM progenitor cells of PD patients likely contributes to inability of AZA monotherapy to restart gene expression and HSPC cycling needed for therapy response. Nevertheless, our study involves plasma targeted metabolite analyses and not the metabolic status of HSPCs isolated from the respective MDS/AML-MRC patients. Therefore, further experimental evidence involving targeted metabolite analysis of the HSPCs is desirable to support conclusions of this study.

To further explain the mechanisms of AZA resistance in PD patients, we observed possible impact of transcriptional dysregulation of other genes already implicated in cancer chemoresistance to deoxycytidine analogues, particularly significant upregulation of *SLC29A1* [32] and downregulation of *NT5C3B* [84] in the PD vs. RD gene set comparisons (Figure S15). In addition, the association of specific mutations with AZA (non)responsiveness may have played a role, given the different proportions of MDS and AML-MRC patients in PD vs. RD patients’ groups (Tables S1 and S2). Although our cohort was not large enough to assess the contribution of these mutations to the AZA response (as mentioned in the Results Section 3.2), the impact of mainly the chromatin remodeling gene mutations present exclusively in the MDS patients (Figure S3) who predominate in the RD group (Tables S1 and S2), cannot be ruled out.

In summary, our data point to the crucial role of quiescent cell-associated cellular metabolism in determining the predisposition to AZA failure in MDS/AML-MRS. From a therapeutic point of view histone deacetylases, which prevent open chromatin occurring at transcriptionally active/poised genes, can be blocked by specific inhibitors [85]. Combinatorial epigenetic-directed therapies of AZA with histone deacetylase inhibitors (HDACi) may be tested in patients with quiescent malignant HSPCs with presumably transcriptionally-nonpermissive chromatin configuration [86]. Previous studies have already documented that HDACi are able to antagonize the quiescent state of LSCs [87] and therefore, AZA and HDACi combinations (already tested in different MDS/AML clinical trials and settings [7,24,26,27,28] may represent an appropriate approach to target malignant stem cells exhibiting signature of pre-existing failure to respond to AZA monotherapy.

## 5. Materials and Methods

### 5.1. CD34^+^ Cells Isolation and RNA Sequencing

BM mononuclear cells (MNCs) were purified using Ficoll-Hypaque density centrifugation (GE Healthcare, Dornstadt, Germany). MNCs were incubated with CD34^+^ magnetic beads (Miltenyi Biotec, Bergisch Gladbach, Germany) and separated using an AutoMACS Pro machine (Miltenyi Biotec) according to the manufacturer’s recommendations. This total (further unsorted) CD34^+^ cell population (referred to as CD34^+^ hematopoietic stem/progenitor cells, HSPCs) was used to prepare RNA. Total RNA was extracted using acid guanidine-thiocyanate-phenol–chloroform method and treated with DNase I (Qiagen, Germantown, MD, USA). RNA quality was assessed using 2100 Bioanalyzer (Agilent, Santa Clara, CA, USA), and RNA integrity numbers were greater than 8 for all samples. cDNA libraries were constructed using NEBNext kit (New England Biolabs, Ipswich, MA, USA). Sequencing was performed on Illumina HiSeq4000 or NovaSeq (Illumina, San Diego, CA, USA).

The raw data have been deposited in the National Center for Biotechnology Information (NCBI) Sequence Read Archive (SRA) database under accession number PRJNA679200.

### 5.2. Mutational Analysis

Mutational screening was performed using the TruSight Myeloid Sequencing Panel (Illumina). The libraries were sequenced on MiSeq or NextSeq instruments (Illumina), and the data were analyzed using NextGENe software (SoftGenetics, State College, PA, USA).

### 5.3. Targeted Metabolic Analysis

Targeted metabolic analysis of blood plasma samples from MDS/AML-MRC patients and age-matched controls were performed using liquid chromatography coupled with tandem mass spectrometry (LC-MS/MS). In total, a concentration of 19 metabolites was measured, including key intermediates of TCA cycle or glycolysis and both enantiomers of 2-hydroxyglutarate (2-HG). Details are described within the Supplementary Materials and Methods section.

### 5.4. IHC of Patients’ Samples

Formalin-fixed and paraffin-embedded tissue samples were processed and prepared for tissue sectioning using the standard protocol for biopsy sample processing. BM trephine biopsies from selected patients were demineralized by 10% chelatone 3 (pH 8) directly after fixation. Visualization of antigens is described in Supplementary Materials and Methods. The primary antibodies used are specified in Table S4.

### 5.5. Bioinformatics and Statistical Analyses

Quantification of gene expression in RNA sequencing data was performed using StringTie2 version 1.3.6 software [88]. Resulting data in the form of a gene count table, were subsequently analyzed and visualized in R software version 4.0.2 (https://www.r-project.org/about.html; Accessed on 7 May 2020) using a variety of packages such as edgeR version 3.30.3 (differential expression analysis), pheatmap version 1.0.12 (heatmaps and dendrograms) and ggplot version 3.3.2 (boxplots, dotplots, barplots, etc.). In some cases, an online tool Heatmapper was used to quickly generate heatmaps to visualize differences in gene expression [89]. Over-representation (ORA) analysis was conducted using an online tool available at ConsensusPathDB website (http://cpdb.molgen.mpg.de; Accessed on 25 September 2020 [33]). GSEA software version 3.0 was used to perform gene set enrichment analyses (GSEA) [90]. All statistical analyses were performed with GraphPad Prism 7 (GraphPad Software, La Jolla, CA, USA) and SPSS software (IBM, Armonk, NY, USA). The statistical tests used to calculate a *p*-value are indicated in legends. A full description of the methods and details of the respective tests are available within the Supplementary Materials and Methods section.

## 6. Conclusions

In this study, we addressed the progenitor cell cycling status and the metabolic pathways that could be correlated with responses to AZA therapy in MDS/AML-MRC patients. Transcriptional signatures (RNA-seq) of AZA pre-treatment CD34^+^ progenitors isolated from MDS/AML-MRC patients, targeted plasma metabolic profiling and selected immunohistochemistry analyses show that AZA-responders have actively cycling progenitors poised for erythro-myeloid differentiation, with high metabolic activity controlling histone acetylation. In contrast, the patients whose condition progressed on AZA therapy revealed CD34^+^ cell cycle quiescence signature, and their progenitors display signs of reduced metabolic activity and impaired metabolically-controlled acetylation of histones needed for transcription-permissive chromatin configuration. We propose that patients be divided into those who are likely to respond to AZA-monotherapy and those who would fail AZA monotherapy, and preferably could be included into trials combining AZA with other agents modulating histone acetylation.

## Data Availability

The raw data have been deposited in the National Center for Biotechnology Information (NCBI) Sequence Read Archive (SRA) database under accession number PRJNA679200.

## Supplementary Materials

The following are available online at https://www.mdpi.com/article/10.3390/cancers13092161/s1, Figure S1: Dendogram obtained from unsupervised hierarchical clustering analysis in patients’ discovery cohort (*n* = 25) and normal controls (*n* = 8), based on normalized RNA-seq expression data, Figure S2: Gene mutational analysis of patients in discovery and validation cohorts, Figure S3: A comparison of mutation frequencies in patients categorized as AML-MRC or MDS within the discovery/validation cohorts, Figure S4: Kaplan–Meier curves of overall survival (OS) and progression-free survival (PFS) in AZA-treated patients divided into three groups according to treatment response status, Figure S5a: Pathways significantly overrepresented in the DEGs between RD and PD, Figure S5b: Pathways significantly overrepresented in the DEGs between RD and CTRL, Figure S5c: Pathways significantly overrepresented in the DEGs between PD and CTRL, Figure S6: Heatmap of the top 50 up and downregulated genes in the PD vs. CTRL samples as identified by GSEA, Figure S7a: Heatmap representation of differential expression of DNA single-strand break repair genes in the CD34^+^ HSPCs from AZA pre-treatment patients (RD, SD, PD) and normal controls (CON), Figure S7b: Heatmap representation of differential expression of DNA double-strand break repair genes in the CD34^+^ HSPCs from AZA pre-treatment patients (RD, SD, PD) and normal controls (CON), Figure S8a: Principal component analysis (PCA) of plasma metabolic profile of the AZA-pre-treatment PD and RD patients, Figure S8b: PCA of plasma metabolic profile of the AZA-pre-treatment PD patients and normal controls, Figure S8c: PCA of plasma metabolic profile of the AZA-pre-treatment RD cohort and normal controls, Figure S9: Heatmap visualization of gene expression profiles for glycolysis pathway genes (*n* = 63, Reactome) in the CD34^+^ HSPCs from AZA pre-treatment patients (RD, SD, PD) and normal controls (CON), Figure S10: Heatmap visualization of gene expression profiles for pyrimidine metabolism genes (*n* = 88, KEGG) in the CD34^+^ HSPCs from AZA pre-treatment patients (RD, SD, PD) and normal controls (CON), Figure S11: Left: Heatmap visualization of DEGs involved in valine/leucine/isoleucine degradation (*n* = 40, KEGG) in the CD34^+^ HSPCs from AZA pre-treatment RD and PD patients. Right: The boxplot graph of relative log2 expression of *BCAT1* in the individual clinical groups (SD, PD, RD) of AZA-pre-treatment patients (*n* = 24) and normal controls (*n* = 8), Figure S12: The boxplot graphs of relative log2 expression of *ACSS1* and *ACSS2* genes in the AZA-pre-treatment HSPCs from patients (*n* = 24) and normal controls (*n* = 8), Student’s *t*-test with Bonferroni correction, *p*-value shown in the graphs, Figure S13: Immunohistochemistry (IHC) staining for IDH1 (a) and pan-acetyl lysine (b) in bone marrow trephine biopsies of MDS/AML-MRC patients, Figure S14: The boxplot graph of relative log2 expression for *SIRT1* gene in the AZA-pre-treatment HSPCs from patients (*n* = 24) and normal controls (*n* = 8), Figure S15: The boxplot graphs of relative log2 expression for *SLC29A1* and *NT5C3B* genes in the AZA-pre-treatment HSPCs from patients (*n* = 24) and normal controls (*n* = 8), Table S1: Characteristics of patients in the discovery cohort, Table S2: Characteristics of patients in the validation cohort, Table S3: Clinical characteristics of all patients in the study, Table S4: Specification of antibodies used in the study, Table S5: The top 10 up and downregulated DEGs between RD and PD. Table S6: Statistical analysis of differences in the levels of selected plasma metabolites between individual clinical groups of AZA-pre-treatment patients and normal controls.

## Appendix A

The discovery cohort included 25 patients diagnosed with MDS (*n* = 20) or AML-MRC with less than 30% myeloblasts in BM (*n* = 5). Their BM specimens (all patients) and peripheral blood plasma samples (11 patients) were obtained during routine clinical assessments at the Institute of Hematology and Blood Transfusion in Prague (IHBT; *n* = 17) and the Department of Hematology, General University Hospital (*n* = 8) in Prague. The validation cohort for plasma metabolites verification consisted of 17 patients with MDS (*n* = 11) and AML-MRC (6) from the IHBT. Collection of samples before first administration of AZA ranged from 0 to 317 days; the time for most patients ranged from 0 to 92 days, only two patient samples were taken 158, and 317 days before AZA administration, respectively. CD34^+^ cells from eight healthy individuals were obtained from Lonza (Basel, Switzerland) and plasma from 16 healthy individuals was used in the validation study. Diagnosis of MDS and AML was made according to the World Health Organization 2016 [91].

An initial response to AZA therapy was assessed using the International Working Group (IWG) response criteria revised in 2006 [92]. The proportion of patients in individual groups from both discovery and validation cohorts was: RD: *n* = 15 (36%), SD: *n* = 13 (31%), PD: (*n* = 14 (33%).

OS was defined as time from start of treatment with AZA to death from any cause or last follow-up. PFS was defined as time from start of treatment until disease relapse/progression or death from any cause. Patients were censored at the time of BMT.

## Appendix B

The correlation analysis between *IDH2* and *BCAT1* expression in CD34^+^ HSPCs from AZA pre-treatment MDS/AML-MRC patients depicted in Figure 4f was created after exclusion of 8 patients from the analysis, i.e., the two *IDH2* mutants, and also *TET2* and *DNMT3A* mutants, according to [54]; the outlier in the correlation, patient V456, showed low level of *IDH2* expression detected by RNA-seq but more abundant IDH2 protein in the IHC assessment, see also Figure 5a.

## Appendix C

Immunophenotypic analysis of CD34^+^ cell population was available for 16 patients of the discovery cohort. The CD34^+^ cell fraction comprised only myeloid blasts in 11/16 cases, in 5/16 cases the CD34^+^ cell fraction comprised myeloid blasts (≥97.5% of total CD34^+^ cells) and precursor B-cells (0.25%, 2.5%, 1.8%, 1% and 1.1% of total CD34^+^ cells, respectively). See also Table S7.
