# Supplementary material for: Low Plasma Citrate Levels and Specific Transcriptional Signatures Associated with Quiescence of CD34+ Progenitors Predict Azacitidine Therapy Failure in MDS/AML Patients
Source: Cancers (Basel). 2021 Apr 30;13(9):2161. doi: 10.3390/cancers13092161 (PMC8125503; doi:10.3390/cancers13092161)
Supplement: Supplementary file 1 [file cancers-13-02161-s001.zip › FINAL_Supplementary Materials and Methods.pdf]

Supplementary Materials and Methods

Bioinformatics and mutational analysis of DNA data

Genomic DNA from BM cells was isolated using the QIAamp DNA Blood Mini Kit (Qiagen Inc., Hilden, Germany). All patients were analyzed for 54 genes recurrently mutated in myeloid disorders using TruSight Myeloid Sequencing Panel (Illumina). Illumina MiSeq and Illumina NextSeq instruments were used to obtain sequence data using V3 300 kit and Mid Output Kit V2, respectively.

Quality of raw data obtained from high-throughput sequencing was checked by FastQC version 0.11.8 [1]. Reads were then trimmed and filtered using Trimmomatic software version 0.39 [2] and resulting files were quality checked by FastQC again. Cleaned up reads obtained by DNA sequencing were mapped to GRCh38 genome using BWA aligner version 0.7.17 [3]. Mapped data was then indexed and sorted using Samtools suite of tools version 1.10 [4].

Variant calling in DNA sequencing data was performed using Freebayes software version 1.3.1 [5]. Discovered variants in form of VCF files were then filtered and annotated using online interface of Ensembl Variant Effect Predictor (VEP) [6]. In the final step, annotated variants were formatted in R software version 4.0.2 (https://www.r-project.org/about.html) and then exported in TSV format.

The interpretation of the clinical significance of genomic variants was verified in several genomic databases (VarSome, dbSNP, PubMed, NCBI, COSMIC, ClinVar, and gnomAD). The minimum of the variant allele frequency (VAF) was set to 5%.

Sample preparation and targeted metabolic analysis

Targeted metabolic analysis of 19 plasma TCA and glycolysis metabolites from MDS/AML-MRC patients (n = 28; 11 from discovery and 17 from validation cohorts) and age-matched controls (n =12) was conducted and used for PCA analyses (**Figure S8a-c**). Concentrations of D-2HG and L-2HG were determined in plasma from the same patients (n = 28) and controls (n = 12). For patients' characterization, 10 TCA and glycolysis metabolites and both enantiomers of 2-HG were selected (**Table S6)**.

All metabolites in plasma were analyzed by LC-MS/MS. Briefly, plasma samples or calibration standards (50 µL) were mixed with 50 µL of phosphate-buffered saline (PBS), 20 µL of internal standard mixture and 480 µL of ice-cold methanol. Calibration standard mixture was prepared in the concentration range 1,000-0 µM by sequential dilution of solutions. Samples were incubated for 1 h at -25 °C and centrifuged at 14,000 × g for 10 min at 4 °C. Supernatants (400 µL) were placed in the new tubes and dried in a centrifugal vacuum concentrator Savant SPD 131 (Fischer Scientific, Pardubice, Czech Republic) and redissolved in 50 µL of water (v/v).

HPLC analysis was performed using a Prominence HPLC system (Shimadzu, Prague, Czech Republic). Sample (1 µL) was injected into ZIC-pHILIC column (50 × 2.1 mm, 5 µm) (Sigma-Aldrich, Prague, Czech Republic) maintained at 35°C. The flow rate was 0.25 mL/min. The mobile phases were composed of 20 mM ammonium acetate, pH 9.0 in 5% acetonitrile (v/v) with medronic acid (5 µM, final concentration) (A) and 20 mM ammonium acetate, pH 9.0 in 90% acetonitrile (v/v) with medronic acid (5 µM, final concentration) (B). Total run time was 15 min. Chromatographic separation of the analytes was performed using a linear gradient as follows: t (min)/% B: 0/100, 6/20, 7.5/20, 8/100. The analytes were detected by mass spectrometer QTRAP 4000 (Sciex, Framingham, MA) working in negative MRM mode using the parameters listed in **Table 1**. The ion source was operated with ion spray voltage set at 4,500 V, curtain gas at 30 psi, ion source temperature at 475 °C, ion source gas 1 and 2 at 33 psi, and collision gas at medium. All MS parameters were optimized by direct infusion; the source parameters by flow injection. Analyst v.1.6 from Sciex was used for the acquisition and analysis of data.

Table 1. QTRAP 4000 mass spectrometer settings for metabolites and their internal standards.

**Metabolite**	**Q1 *m/z***	**Q3 *m/z***	**DP [V]**	**CE [V]**	**CXP [V]**
Citrate	190.8	110.7	-45	-18	-7
Isocitrate	190.9	154.8	-35	-18	-7
Malate	132.8	114.7	-35	-16	-5
α-Ketoglutarate	145.0	100.8	-30	-12	-5
Lactate	88.9	42.8	-45	-20	-5
Pyruvate	86.9	42.9	-35	-12	-5
Succinate	116.8	73,0	-45	-18	-5
Glycerate	104.8	74.7	-35	-14	-1
Glycolate	74.9	46.8	-45	-14	-7
Fumarate	114.8	70.8	-35	-12	-1
Taurin	123.9	79.8	-25	-28	-3
3-Hydroxybutyrate	102.9	58.9	-35	-16	-9
Urate	166.9	123.8	-40	-22	-7
cis-Aconitate	172.9	84.9	-30	-18	-3
3-Phosphoglycerate	184.9	96.8	-45	-22	-15
Phosphoethanolamine	139.9	78.8	-50	-22	-3
Pyroglutamate	127.8	83.9	-60	-16	-3
Phosphoenolpyruvate	166.9	78.9	-30	-18	-5
Allantoin	156.7	113.7	-45	-16	-5
**Internal standard**	**Q1 *m/z***	**Q3 *m/z***	**DP [V]**	**CE [V]**	**CXP [V]**
Pyruvate D4	89.8	44.9	-35	-12	-5
Lactate 13C3	91.8	44.9	-20	-24	-1
Fumarate 13C4	118.8	73.8	-40	-12	-1
Succinate 13C4	120.8	75.8	-40	-16	-1
Malate D3	135.8	116.8	-40	-18	-5
α-Ketoglutarate D6	148.8	104.8	-30	-12	-5
Citrate 13C6	196.8	115.7	-45	-18	-5
Allantoin 5-13C, 1-15N	158.8	98.8	-50	-22	-7

Abbreviations: IS - internal standard; Q: quadrupole; DP: declustering potential; CE: collision energy; CXP: collision cell exit potential.

D/L-enantiomers of 2-HG were analyzed essentially according to Poinsignon et al. [7]. Plasma samples or calibration standard (50 μL) was mixed with 10 μL of internal standard (D-2-HG 13C5; 5 μg/mL) and 600 μL water. Calibration standards (D and L-2-HG) were prepared in the range 20,000-0 ng/mL by sequential dilution of solutions. Samples were processed by solid phase extraction (Strata SPE 30, Phenomenex, Torrance, CA). Eluted analytes were dried at 80 °C in a centrifugal vacuum concentrator Savant SPD 131 (Fischer Scientific, Pardubice, Czech Republic). To separate the D/L-enantiomers, dried samples were derivatized by (+)-O,O′-diacetyl-l-tartaric anhydride (DATAN) solution in methylene chloride − acetic acid (4:1, v/v), at 80 °C for 40 min. Samples were dried at 50 °C and then dissolved in 40 μL of 10 mM ammonium formate pH 3.2 in 50% acetonitrile (v/v).

HPLC analysis was performed using a Prominence HPLC system (Shimadzu, Prague, Czech Republic). Sample (1μL) was injected into ZIC-HILIC column (150 × 2.1 mm, 5 μm) (Merck, Darmstadt, Germany)

maintained at 30 °C. The flow rate was 0.20 mL/min. The DATAN derivatized enantiomers were separated by isocratic elution using 10 mM ammonium formate pH 3.2 in 78% acetonitrile (v/v). Total run time was 8 min. The analytes were detected by mass spectrometer QTRAP 4000 (Sciex, Framingham, MA) working in negative MRM mode using the parameters listed in **Table 2**. The ion source was operated with ion spray voltage set at 4,500 V, curtain gas at 30 psi, ion source temperature at 500 °C, ion source gas 1 and 2 at 40 resp. 50 psi, and collision gas at medium. All MS parameters were optimized by direct infusion; the source parameters, by flow injection. Analyst v.1.6 from Sciex was used for the acquisition and analysis of data.

Table 2. QTRAP 4000 mass spectrometer settings for L/D-2-HG and internal standard DATAN derivatives.

**Analyte, DATAN derivative**	**Q1 *m/z***	**Q3 *m/z***	**DP [V]**	**CE [V]**	**CXP [V]**
L-2-Hydroxyglutarate	363.0	146.8	-35	-12	-7
D-2-Hydroxyglutarate	363.1	146.8	-35	-12	-7
D-2- Hydroxyglutarate 13C5	368.1	151.9	-35	-12	-9

Abbreviations: IS - internal standard; Q: quadrupole; DP: declustering potential; CE: collision energy; CXP: collision cell exit potential, DATAN: (+)-O,O′-diacetyl-l-tartaric anhydride.

Bioinformatics and statistical analyses of RNA data

Quality of raw data obtained from high-throughput sequencing was checked by FastQC version 0.11.8 [1]. Reads were then trimmed and filtered using Trimmomatic software version 0.39 [2] and resulting files were quality checked by FastQC again. Cleaned up data from RNA sequencing was then mapped to GRCh38 version of human genome using STAR aligner version 2.7.2b [8]. Mapped data was then indexed and sorted using Samtools suite of tools version 1.10 [4].

Differential expression analysis (DEA) was performed in R software, using edgeR version 3.30.3 package according to relevant manuals [9,10]. All input expression data were normalized, using quantile normalization, also provided by edgeR package. Normalized data were subsequently used to create heatmaps (pheatmap version 1.0.12), boxplots (ggplot version 3.3.2) and other graphical representations in this work. Student's t-tests used in boxplots were performed in R software using rstatix package version 0.6.0, according to standard package manuals. Hierarchical clustering analysis of normalized expression data was performed using "dist" and "hclust" functions, according to standard package manuals (mentioned functions are part of R software stats package version 3.6.2). Results of hierarchical clustering were then displayed in form of dendrogram using basic "plot" function in R software.

Differences in the distribution of continuous variables between categories were analyzed by either Mann-Whitney or Kruskal–Wallis test. Patients' groups with nominal variables were compared by chi-square test. Survival analysis was considered from the date of start of AZA treatment to date of death or last contact. Survival curves were prepared by the Kaplan–Meier method and compared by the log-rank test. All statistical analyses were performed using GraphPad Prism 7 (GraphPad Software, La Jolla, CA) and SPSS software (IBM, Armonk, NY).

Pathway over-representation analysis and GSEA

Over-representation (ORA) analysis was performed using online tool available at ConsensusPathDB website (http://cpdb.molgen.mpg.de; [11]). Based upon results from differential expression analysis (DEA) performed using edgeR version 3.30.3 (R software), a list of significantly deregulated genes (FDR < 0.05) was uploaded to ConsensusPathDB online platform. ORA analysis was performed using "Pathway-based

sets" option, with all available datasets enabled. Minimum overlap of pathway with input list was set to 15 genes and p-value cutoff was set to 0.01. Resulting data table was downloaded and further processed in R software. Adjusted p-value was transformed using negative logarithm with base 10 for easier implementation in plots.

Gene set enrichment analyses (GSEA) were performed with GSEA software version 3.0 [12]. GSEA was used in two variations: full data GSEA and pre-ranked GSEA. First approach required full count data table as input resource and was therefore able to generate gene expression heatmaps alongside standard GSEA plots and tables. In case of pre-ranked GSEA, a list of significantly deregulated genes with their respective logarithmic fold changes, obtained from DEA previously performed in R software, was inputted into analysis. To identify possible gene sets and pathways of interest, two major collections (H and C2) and one sub-collection (C5: GO Biological Process) of gene sets were chosen from Molecular Signatures Database (https://www.gsea-msigdb.org/gsea/msigdb/index.jsp), and then used in preliminary GSEA analyses. Custom built collection of relevant gene sets was later used in GSEA analyses, to reduce computational strain of analyses and to facilitate focus on specific pathways.

Principal Component Analysis

In order to preserve homogeneity within the groups of RD and PD patients from two different cohorts used for targeted metabolic analysis, the patients' datasets and controls were verified through principal component analysis (PCA) using MetaboAnalyst online tool version 5.0 [13].

Immunohistochemistry of patients' samples - visualization of antigens

Antigen unmasking was done in the microwave histoprocessor (Histos 3, Milestone, Shelton, CT) by 10 mM citrate buffer, pH 6.0, 120 °C, using high pressure, for 15 min with gradual cooling. The following detection was used: Mouse/Rabbit PolyDetector DAB HRP Brown Detection System (Bio SB, Santa Barbara, CA). DAB chromogen (included in the detection system) was used for visualization of reaction. All washing steps were made twice in 0.5 M Tris-HCl, pH 7.6 (5 min each) and once in 0.5 M Tris-HCl, pH 7.6 with 0.5% Tween 20 (5 min). Finally, cell nuclei were counterstained by hematoxylin and the slide was coverslipped. In double staining process, identical detection system was used and desired antigens were stained sequentially. The cytoplasmic/membranous protein (CD34) was stained before the nuclear marker (H3K9ac). The antigens were visualized using Envision FLEX HRP Magenta Substrate Chromogen System (Agilent, Santa Clara, CA) (first primary antibody) and the DAB chromogen (second primary antibody).

Flow cytometric detection of $CD34^+$ cells and their myeloid and B-lymphoid subsets in BM samples

Evaluation of MDS by flow cytometry was performed in accordance with the ELN recommendations [14,15]. Briefly, following bulk lysis with NH4Cl, bone marrow cells were stained with appropriate antibody combinations allowing the phenotypic analysis of myeloid, B-lymphoid and erythroid precursors as well as lymphoid, monocytic and neutrophilic cell compartments. Acquisition and analysis were performed on BD FACSCanto II (BD Biosciences) cytometer equipped with blue, red and violet lasers. For the analysis of precursor cells, a minimum of 100 000 $CD45^+$ events and preferably 500 $CD34^+$ events were acquired. Myeloid precursors were defined based on CD45dim, $CD13^+$, $CD33^+$, $CD34^+$, $CD117^+$, HLA-DR^+ and B-lymphoid precursors were defined based on CD45dim, $CD10^+$, $CD19^+$, $CD34^+$ $CD20^-$.

References:

1. Andrews, S. A Quality Control Tool for High Throughput Sequence Data.
2. Bolger, A.M.; Lohse, M.; Usadel, B. Trimmomatic: A Flexible Trimmer for Illumina Sequence Data. *Bioinformatics* **2014**, *30*, 2114–2120, doi:10.1093/bioinformatics/btu170.
3. Li, H.; Durbin, R. Fast and Accurate Short Read Alignment with Burrows-Wheeler Transform. *Bioinformatics* **2009**, *25*, 1754–1760, doi:10.1093/bioinformatics/btp324.
4. Li, H.; Handsaker, B.; Wysoker, A.; Fennell, T.; Ruan, J.; Homer, N.; Marth, G.; Abecasis, G.; Durbin, R.; 1000 Genome Project Data Processing Subgroup The Sequence Alignment/Map Format and SAMtools. *Bioinformatics* **2009**, *25*, 2078–2079, doi:10.1093/bioinformatics/btp352.
5. Garrison, E.; Marth, G. Haplotype-Based Variant Detection from Short-Read Sequencing. *eprint arXiv:1207.3907 2012*.
6. Yates, A.D.; Achuthan, P.; Akanni, W.; Allen, J.; Allen, J.; Alvarez-Jarreta, J.; Amode, M.R.; Armean, I.M.; Azov, A.G.; Bennett, R.; et al. Ensembl 2020. *Nucleic Acids Research* **2019**, gkz966, doi:10.1093/nar/gkz966.
7. Poinsignon, V.; Mercier, L.; Nakabayashi, K.; David, M.D.; Lalli, A.; Penard- Lacronique, V.; Quivoron, C.; Saada, V.; De Botton, S.; Broutin, S.; et al. Quantitation of Isocitrate Dehydrogenase (IDH)-Induced D and L Enantiomers of 2-Hydroxyglutaric Acid in Biological Fluids by a Fully Validated Liquid Tandem Mass Spectrometry Method, Suitable for Clinical Applications. *Journal of Chromatography B* **2016**, *1022*, 290–297, doi:10.1016/j.jchromb.2016.04.030.
8. Dobin, A.; Davis, C.A.; Schlesinger, F.; Drenkow, J.; Zaleski, C.; Jha, S.; Batut, P.; Chaisson, M.; Gingeras, T.R. STAR: Ultrafast Universal RNA-Seq Aligner. *Bioinformatics* **2013**, *29*, 15–21, doi:10.1093/bioinformatics/bts635.
9. Kovaka, S.; Zimin, A.V.; Pertea, G.M.; Razaghi, R.; Salzberg, S.L.; Pertea, M. Transcriptome Assembly from Long-Read RNA-Seq Alignments with StringTie2. *Genome Biology* **2019**, *20*, doi:10.1186/s13059-019-1910-1.
10. Robinson, M.D.; McCarthy, D.J.; Smyth, G.K. EdgeR: A Bioconductor Package for Differential Expression Analysis of Digital Gene Expression Data. *Bioinformatics* **2010**, *26*, 139–140, doi:10.1093/bioinformatics/btp616.
11. Herwig, R.; Hardt, C.; Lienhard, M.; Kamburov, A. Analyzing and Interpreting Genome Data at the Network Level with ConsensusPathDB. *Nature Protocols* **2016**, *11*, 1889–1907, doi:10.1038/nprot.2016.117.
12. Subramanian, A.; Tamayo, P.; Mootha, V.K.; Mukherjee, S.; Ebert, B.L.; Gillette, M.A.; Paulovich, A.; Pomeroy, S.L.; Golub, T.R.; Lander, E.S.; et al. Gene Set Enrichment Analysis: A Knowledge-Based Approach for Interpreting Genome-Wide Expression Profiles. *Proceedings of the National Academy of Sciences* **2005**, *102*, 15545–15550, doi:10.1073/pnas.0506580102.
13. Chong, J.; Wishart, D.S.; Xia, J. Using MetaboAnalyst 4.0 for Comprehensive and Integrative Metabolomics Data Analysis. *Current Protocols in Bioinformatics* **2019**, *68*, doi:10.1002/cpbi.86.
14. van de Loosdrecht, A.A.; Alhan, C.; Bene, M.C.; Della Porta, M.G.; Drager, A.M.; Feuillard, J.; Font, P.; Germing, U.; Haase, D.; Homburg, C.H.; et al. Standardization of Flow Cytometry in Myelodysplastic Syndromes: Report from the First European LeukemiaNet Working Conference on Flow Cytometry in Myelodysplastic Syndromes. *Haematologica* **2009**, *94*, 1124–1134, doi:10.3324/haematol.2009.005801.
15. Westers, T.M.; Ireland, R.; Kern, W.; Alhan, C.; Balleisen, J.S.; Bettelheim, P.; Burbury, K.; Cullen, M.; Cutler, J.A.; Della Porta, M.G.; et al. Standardization of Flow Cytometry in Myelodysplastic Syndromes: A Report from an International Consortium and the European LeukemiaNet Working Group. *Leukemia* **2012**, *26*, 1730–1741, doi:10.1038/leu.2012.30.
