# Supplementary material for: Low Plasma Citrate Levels and Specific Transcriptional Signatures Associated with Quiescence of CD34+ Progenitors Predict Azacitidine Therapy Failure in MDS/AML Patients
Source: Cancers (Basel). 2021 Apr 30;13(9):2161. doi: 10.3390/cancers13092161 (PMC8125503; doi:10.3390/cancers13092161)
Supplement: Supplementary file 1 [file cancers-13-02161-s001.zip › FINAL_Supplementary_Figures_Tables.pdf]

**Table S1:** Characteristics of patients in the discovery cohort

| Patient ID | Response | Sex | Age (yrs) | WHO class. | Mutations (VAF %) | Karyotype | BM blasts (%) | Hb (g/L) | ANC (×10⁹/L) | Platelet count (×10⁹/L) | IPSS-R score in MDS | IPSS-R in MDS |
|---|---|---|---|---|---|---|---|---|---|---|---|---|
| V1788 | RD | M | 81 | MDS-EB-2 | *TP53* (59)<br>*SF3B1* (40) | Complex | 13.6 | 66 | 0.22 | 78 | 9.5 | very high |
| V1456 | RD | F | 75 | MDS-MLD | *SF3B1* (39)<br>*TET2* (46)<br>*CUX1*(49) | 46,XX[1],<br>46,XX,idic(X)(q13)[8],<br>46,XX,idic(X)(q13),<br>+idic(X)(q13) [20],<br>46,XX,idic(X)(q13),<br>+idic(X)(q13), +idic(X)(q13)[1] | 7 | 88 | 0.31 | 27 | 6.5 | very high |
| V839 | RD | F | 78 | MDS-EB-2 | *DNMT3A* (44)<br>*RUNX1* (26) | 46,XX[3];<br>47,XX,del(5)(q31),+8[19] | 7.7 | 88 | 1.97 | 90 | 4.5 | intermediate |
| V406 | RD | M | 67 | MDS-EB-2 | *SF3B1* (27) | 46,XY[8];<br>47,XY,del(5)(q31),+8[14] | 18 | 89 | 0.61 | 74 | 6 | high |
| V574 | RD | F | 77 | AML-MRC | *JAK2* (41) | 46,XX[5];<br>46,XX,del(5)(q31.2q33.1)[18] | 24 | 109 | 11.80 | 578 | NA | NA |
| V538 | RD | F | 82 | MDS-EB-1 | *DNMT3A*(46)<br>*BCOR* (40) | 46,XX[5];<br>46,XX,del(5)(q22q32.2)[17] | 4.6 | 107 | 3.27 | 124 | 2 | low |
| V624 | RD | F | 74 | MDS-EB-2 | *SF3B1*(42)<br>*TP53*(47)<br>*RUNX1* (42) | 46,XX,del(5)(q13.2q34)[6] | 18 | 82 | 0.40 | 80 | 6 | high |
| V1884 | RD | F | 73 | MDS-EB-2 | *TP53* (36)<br>*TP53*(40) | Complex | 18.2 | 91 | 0.34 | NA | 9.5 | very high |
| V655 | RD | F | 63 | MDS-EB-2 | *TP53* (69) | Complex | 15.6 | 95 | 0.44 | 65 | 9 | very high |
| V1279 | RD | M | 65 | MDS-EB-2 | *TET2* (19)<br>*TET2* (22)<br>*EZH2* (6)<br>*ZRSR2* (68) | 46,XY[22] | 11 | 117 | 0.24 | 172 | 4.5 | intermediate |
| V716 | SD | M | 63 | MDS-EB-2 | *SF3B1*(18) | 46,XY[1];<br>45,XY,del(5)(q12q33.3),<br>dic(7;11)(p12;q13)[23] | 14.2 | 117 | 2.68 | 51 | 4.5 | intermediate |
| V1874 | SD | M | 74 | AML-MRC | *0* | 46,XY[22] | 27.4 | 127 | 1.32 | 81 | NA | NA |
| V1441 | SD | F | 70 | MDS-EB-2 | *RUNX1* (30)<br>*TET2* (6)<br>*BCOR* (28) | 46,XX, t(9;21) [14] | 10.2 | 86 | 0.54 | 53 | 5 | high |

| | | | | | | | | | | | | |
|---|---|---|---|---|---|---|---|---|---|---|---|---|
| V1337 | SD | M | 44 | MDS-EB-2 | *WT1* (7)<br>*TP53* (3) | 46,XY[16],<br>46,XY,t(3;21)(q11.2;q22),<br>del(7)(q22)[5] | 6.6 | 127 | 1.82 | 70 | 3.5 | intermediate |
| V1394 | SD | M | 69 | MDS-EB-2 | *NRAS* (46)<br>*ETV6* (49)<br>*ASXL1* (38)<br>*STAG2* (90)<br>*PHF6* (96)<br>*GATA2* (43) | 46,XY[20] | 12.2 | 101 | 3.71 | 37 | 5 | high |
| V1592 | SD | F | 64 | MDS-EB-2 | *TET2* (45)<br>*RUNX1* (45)<br>*ASXL1* (50)<br>*EZH2* (49)<br>*PHF6* (45) | 46,XX[5]; 47,XX,+8[20] | 13 | 100 | 4.97 | 26 | 6 | high |
| V456 | PD | F | 68 | MDS-EB-2 | *0* | 46,XX[15] | 18.4 | 90 | 1.15 | 141 | 5 | high |
| V1857 | PD | F | 73 | MDS-EB-2 | *DNMT3A* (41) | 46,XX[22] | 17 | 111 | 0.93 | 20 | 5.5 | high |
| V714 | PD | M | 70 | AML-MRC | *CBL* (8)<br>*NRAS*(8) | 46,XY[13] | 27 | 91 | 0.06 | 34 | NA | NA |
| V777 | PD | M | 68 | AML-MRC | *0* | 46,XY[14] | 27 | 106 | 1.73 | 106 | NA | NA |
| V637 | PD | M | 65 | MDS-EB-2 | *0* | 47,XY,+11[22] | 10 | 83 | 0.39 | 80 | 6 | high |
| V712 | PD | M | 67 | MDS-EB-2 | *SRSF2* (32)<br>*ASXL1* (26)<br>*RUNX1* (30)<br>*BCOR* (6)<br>*STAG2* (6)<br>*STAG2* (5) | 46,XY[22] | 19.4 | 97 | 0.41 | 45 | 6.5 | very high |
| V1297 | PD | M | 68 | MDS-EB-2 | *TP53* (8) | Complex | 10.6 | 98 | 0.26 | 38 | 8.5 | very high |
| V1554 | PD | M | 62 | AML-MRC | *IDH2* (5)<br>*IKZF1* (22)<br>*STAG2* (6) | 46,XY [22],<br>46,XY,del(5)(q13q33) [2] | 26 | 91 | 1.02 | 124 | NA | NA |
| V344 | PD | F | 64 | MDS-EB-2 | *TP53* (68)<br>*SF3B1* (40)<br>*DNMT3A* (52) | 46,XX[13],del(5)(q15q33.3)[9] | 17.2 | 91 | 3.56 | 35 | 6 | high |

VAF – variant allele frequency, BM – bone marrow, Hb – hemoglobin, WHO class. – WHO classification, NA – not available

**Table S2:** Characteristics of patients in the validation cohort

| Patient ID | Response | Sex | Age (yrs) | WHO class. | Mutations (VAF %) | Karyotype | BM blasts (%) | Hb (g/L) | ANC (×10⁹/L) | Platelet count (×10⁹/L) | IPSS-R score in MDS | IPSS-R in MDS |
|---|---|---|---|---|---|---|---|---|---|---|---|---|
| P1618 | RD | F | 65 | MDS-MLD | *CSF3R* (22)<br>*DNMT3A* (33)<br>*TP53* (30)<br>*U2AF1* (28) | No metaphases (FISH -7) | 2.6 | 107 | 0.85 | 33 | 5 | high |
| P1648 | RD | M | 75 | MDS-EB-1 | *DNMT3A* (9) | NA | 9,2 | 129 | 2.12 | 95 | NA | NA |
| P942 | RD | F | 78 | AML-MRC | *DNMT3A* (41) | NA | 7.2 | 77 | 1,91 | 120 | NA | NA |
| P1040 | RD | M | 62 | MDS-EB-2 | *0* | 46,XY[22] | 12.8 | 115 | 1,53 | 176 | 4 | intermediate |
| P2291 | RD | F | 68 | AML-MRC | *TP53* (52) (8)<br>*TET2* (37) | Complex | 8.2 | 91 | 2.03 | 72 | NA | NA |
| P1605 | SD | F | 76 | AML-MRC | *IDH2* (26)<br>*DNMT3A* (50) | 46,XX[8] | 41 | 86 | 0.1 | 76 | NA | NA |
| P1775 | SD | M | 71 | MDS-EB-2 | *PTEN* (10) | 46,XY[22] | 12.6 | 137 | 1.68 | 84 | 4.5 | intermediate |
| P1824 | SD | M | 71 | MDS-EB-2 | *ASXL1 (25)*<br>*RUNX1*(11)<br>*SF3B1 (42)*<br>*ZRSR2 (25)*<br>*STAG2* (35) | 46,XY[17] | 6.6 | 88 | 1.59 | 216 | 5 | high |
| P1898 | SD | M | 80 | MDS EB-2 | *ASXL1* (6) | 46,XY[6] | 11 | 109 | 0.31 | 63 | 5 | high |
| P1836 | SD | M | 40 | MDS-EB-2 | *0* | 46,XY[6] | 19.6 | 91 | 0.26 | 23 | 6.5 | very high |
| P1357 | SD | F | 72 | MDS-EB-1 | *TP53* (18) | Complex | 8.6 | 75 | 0.53 | 49 | 9 | very high |
| P683 | SD | M | 56 | MDS-EB-2 | *DNMT3A* (39)<br>*JAK2* (50) | 46,XY[22] | 10.2 | 84 | 4.41 | 122 | 5 | high |
| P1371 | PD | F | 69 | AML-MRC | *0* | 46, XX [7] | 26.6 | 119 | 0.77 | 258 | NA | NA |
| P1564 | PD | M | 72 | AML-MRC | *TP53* (15) | Complex | 13.8 | 77 | 0.36 | 29 | NA | NA |
| P1803 | PD | M | 71 | MDS-EB-2 | *DNMT3A* (41) | Complex | 12.8 | 79 | 2.36 | 34 | 5.5 | high |
| P689 | PD | F | 65 | AML-MRC | *DNMT3A* (42*)*<br>*RUNX1* (33)<br>*U2AF1* (38) | No metaphases | 15.2 | 87 | 0.5 | 144 | NA | NA |

| | | | | | | | | | | | | |
|---|---|---|---|---|---|---|---|---|---|---|---|---|
| P1333 | PD | M | 66 | MDS-EB-1 | *SETBP1* (24) *DNMT3A* (26) | No metaphases (FISH-7) | 7.4 | 77 | 0.39 | 72 | 7.5 | very high |

VAF – variant allele frequency, BM – bone marrow, Hb – hemoglobin, WHO class. – WHO classification, NA – not available

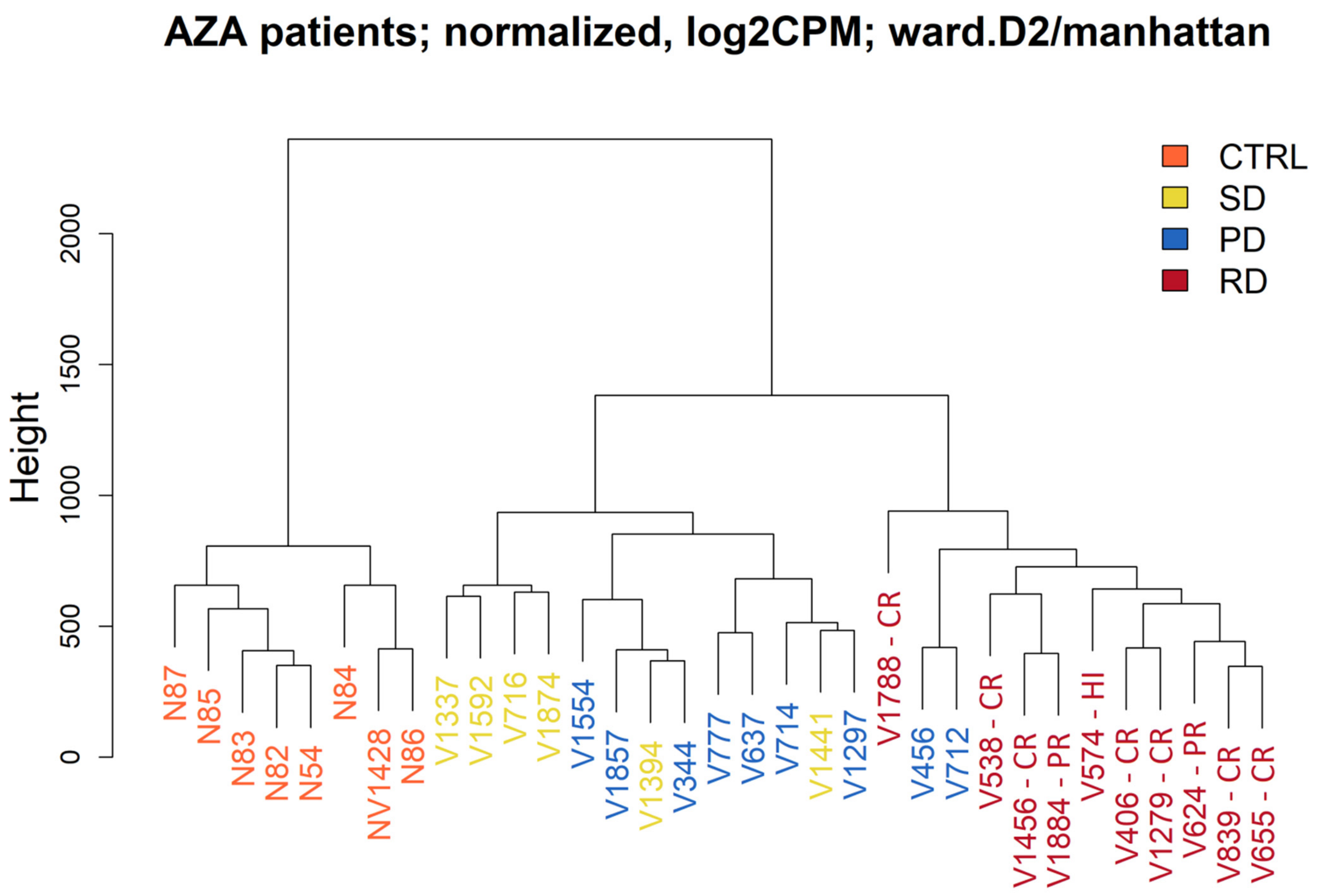


**Figure S1:** Dendogram obtained from unsupervised hierarchical clustering analysis in patients' discovery cohort (n = 25) and normal controls (n = 8), based on normalized RNA-seq expression data (details are described in the Supplementary Materials and Methods section). Unsupervised hierarchical clustering separated the samples into two main groups, patients and controls. Within the patients' group, the branch of RD patients contains grouped patients with complete remission (CR), partial responses (PR) and hematological improvements (HI).

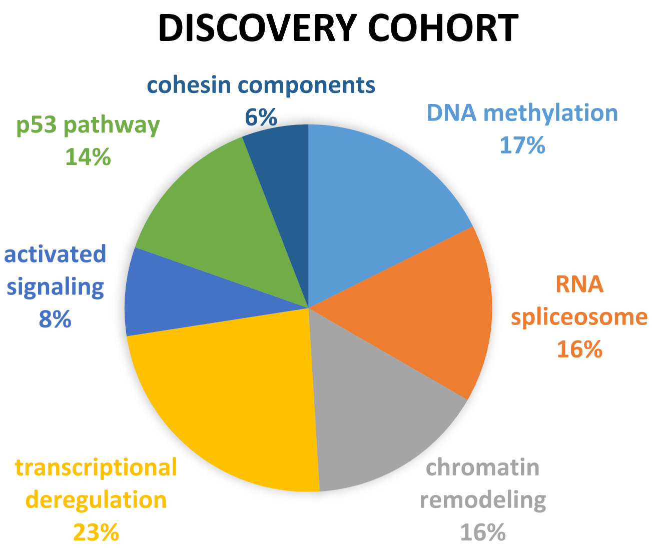


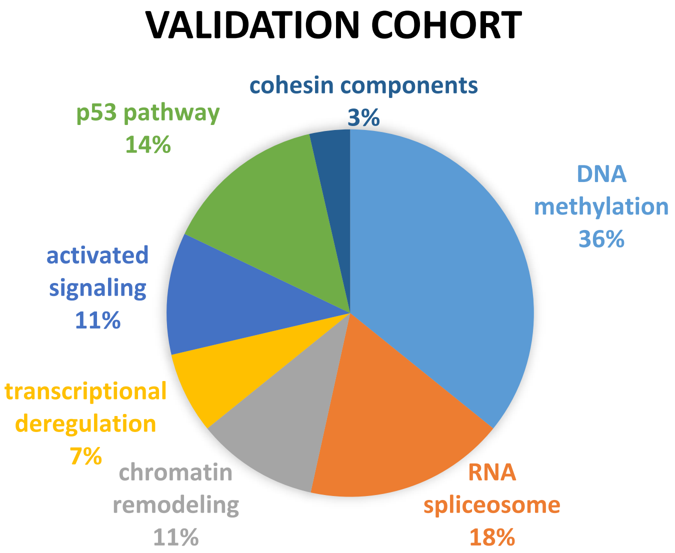


| Functional group (frequency, %) | Total (n=42) | Discovery cohort (n=25) | Validation cohort (=17) | *P* value |
|---|---|---|---|---|
| **DNA methylation (*DNMT3A, TET2, IDH1/2*)** | 19 (45%) | 9 (36%) | 10 (59%) | 0.253 |
| **RNA spliceosome (*SF3B1, SRSF2, ZRSR2, U2AF1*)** | 12 (29%) | 8 (32%) | 5 (29%) | 0.804 |
| **Chromatin remodeling (*ASXL1, EZH2, BCOR, SETBP1*)** | 11 (26%) | 8 (32%) | 3 (18%) | 0.496 |
| **Transcriptional deregulation (*RUNX1, WT1, ETV6, CUX1, GATA2, PHF6, IKZF1*)** | 14 (33%) | 12 (48%) | 2 (12%) | 0.010 |
| **Activated signaling (*NRAS, CBL, JAK2, PTEN, CSF3R*)** | 7 (17%) | 4 (16%) | 3 (18%) | 0.779 |
| **p53 pathway (*TP53*)** | 11 (26%) | 7 (28%) | 4 (24%) | 0.973 |
| **Cohesin components (*STAG2*)** | 4 (10%) | 3 (12%) | 1 (6%) | 0.899 |

**Figure S2:** Gene mutational analysis of patients in discovery and validation cohorts. Mutation frequencies are categorized according to functional classification. For mutation analysis methodology see Supplementary Materials and Methods. P of the analysis is the p value of the chi-square test with Yates' correction for small sample sizes.

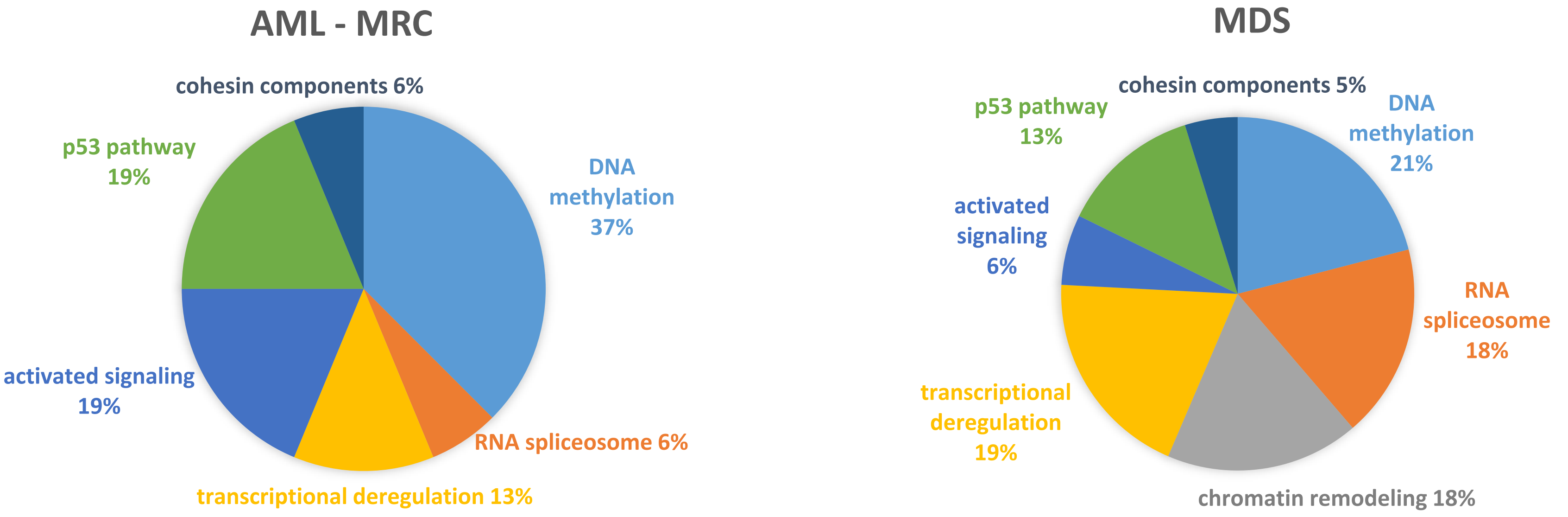


**Figure S3:** A comparison of mutation frequencies in patients categorized as AML-MRC or MDS within the discovery/validation cohorts. Mutation frequencies are categorized according to the functional classification of respective genes, as described in Figure S2 above.

**Table S3:** Clinical characteristics of all patients in the study

| Parameter | Responders | Stable disease | Progressive disease | p-value[a] |
|---|---|---|---|---|
| Total, n | 15 | 13 | 14 | |
| Age (yrs), median (range) | 74 (62-82) | 70 (40-80) | 68 (62-73) | 0.14 |
| Patients sex | | | | 0.13 |
| Male (%) | 5 (33) | 9 (69) | 9 (64) | |
| Female (%) | 10 (67) | 4 (31) | 5 (36) | |
| Bone marrow blasts (%), median (range) | 11.0 (2.6-24) | 12.2 (6.6-30) | 17.1 (7.4-27) | 0.08 |
| Hemoglobin (g/dL), median (range) | 9.1 (6.6-12.9) | 10 (7.5-13.7) | 9.1 (7.7-11.9) | 0.56 |
| Platelet count ($\times10^9$/L), median (range) | 85 (27-578) | 93 (23-216) | 59 (20-258) | 0.27 |
| ANC ($\times10^9$/L), median (range) | 0.9 (0.2-11.8) | 1.6 (0.1-5.0) | 0.5 (0.1-3.56) | 0.48 |
| Cytogenetic risk[b] (%) | | | | 0.51 |
| Very good, Good | 6 | 10 | 8 | |
| Intermediate | 2 | 1 | 1 | |
| Poor, Very poor | 4 | 2 | 3 | |
| Not applicable | 3 | 0 | 2 | |
| Number of mutations, median (range) | 2 (0-6) | 2 (0-6) | 1 (0-6) | 0.47 |
| WHO classification, n | | | | 0.31 |
| MDS-MLD | 2 | 0 | 0 | |
| MDS-EB1 | 2 | 1 | 1 | |
| MDS-EB2 | 8 | 10 | 7 | |
| AML-MRC | 3 | 2 | 6 | |
| OS since start AZA, median (months) | 22.9 | 47.0 | 7.6 | <0.0001 |
| PFS since start AZA, median (months) | 20.8 | 47.0 | 3.1 | <0.0001 |

[a] p-values were calculated by Kruskal-Wallis test for continuous variables, Fisher's exact test for categorical variables and Log Rank (Mantel-Cox) test for survival analysis.

[b] The cytogenetic risk was calculated based on the IPSS-R cytogenetic risk groups for MDS and were used also for AML-MRC.

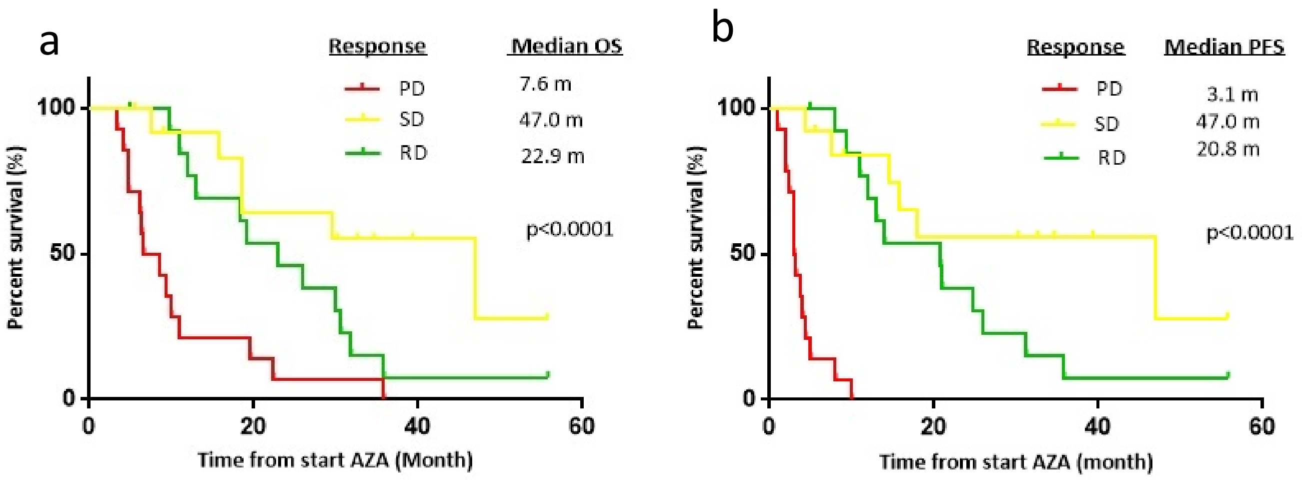


**Figure S4:** Kaplan–Meier curves of overall survival (OS) and progression-free survival (PFS) in AZA – treated patients divided into three groups according to treatment response status. Patients of discovery and validation cohorts were grouped into progressive disease (PD, n=14), stable disease (SD, n=13) and responders (RD, n=15) by response status. (A) OS in PD, SD and RD patients was 7.6, 47.0 and 22.9 months, respectively (*P* <0.0001). (B) PFS in PD, SD and RD patients was 3.1, 47.0 and 20.8 months, respectively (*P* <0.0001).

**Table S4:** Specification of antibodies used in the study.

| ANTIGEN | HOST | CLONALITY | CLONE | DILUTION | PRODUCT N. | COMPANY |
|---|---|---|---|---|---|---|
| IDH1 | Rabbit | poly | - | 1:1000 | ab94571 | Abcam |
| IDH2 | Rabbit | mono | EPR7577 | 1:1000 | ab131263 | Abcam |
| pan-acetyl-lysine | Rabbit | poly | - | 1:200 | ab80178 | Abcam |
| AIF | Rabbit | mono | E20 | 1:250-500 | ab32516 | Abcam |
| H3K9acetyl | Rabbit | mono | C5B11 | 1:1500 | 9649 | Cell Signaling |
| $CD34^{+}$ | Mouse | mono | QBEnd-10 | 1:50 | M7165 | Dako |

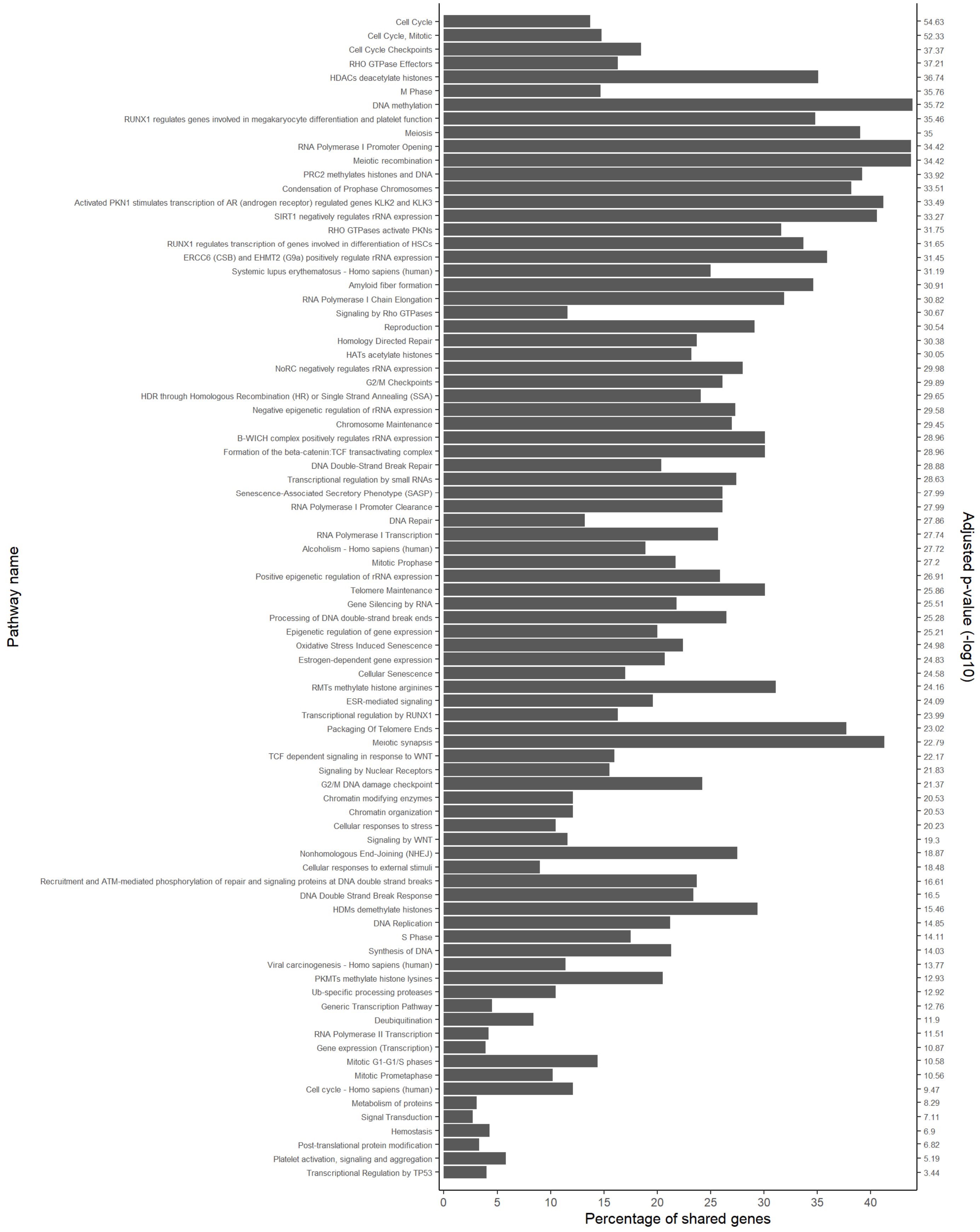


**Figure S5a:** Pathways significantly overrepresented in the DEGs between RD and PD. Over-representation analysis (ORA) made by ConsensusPathDB using DEGs (FDR <0.05, FC >0) with minimum overlap of 15 genes in pathway and p-value cut-off of 0.01. The percentage of DEGs associated with each biological process is shown along the x-axis and -log10-transformed p-values on the y-axis.

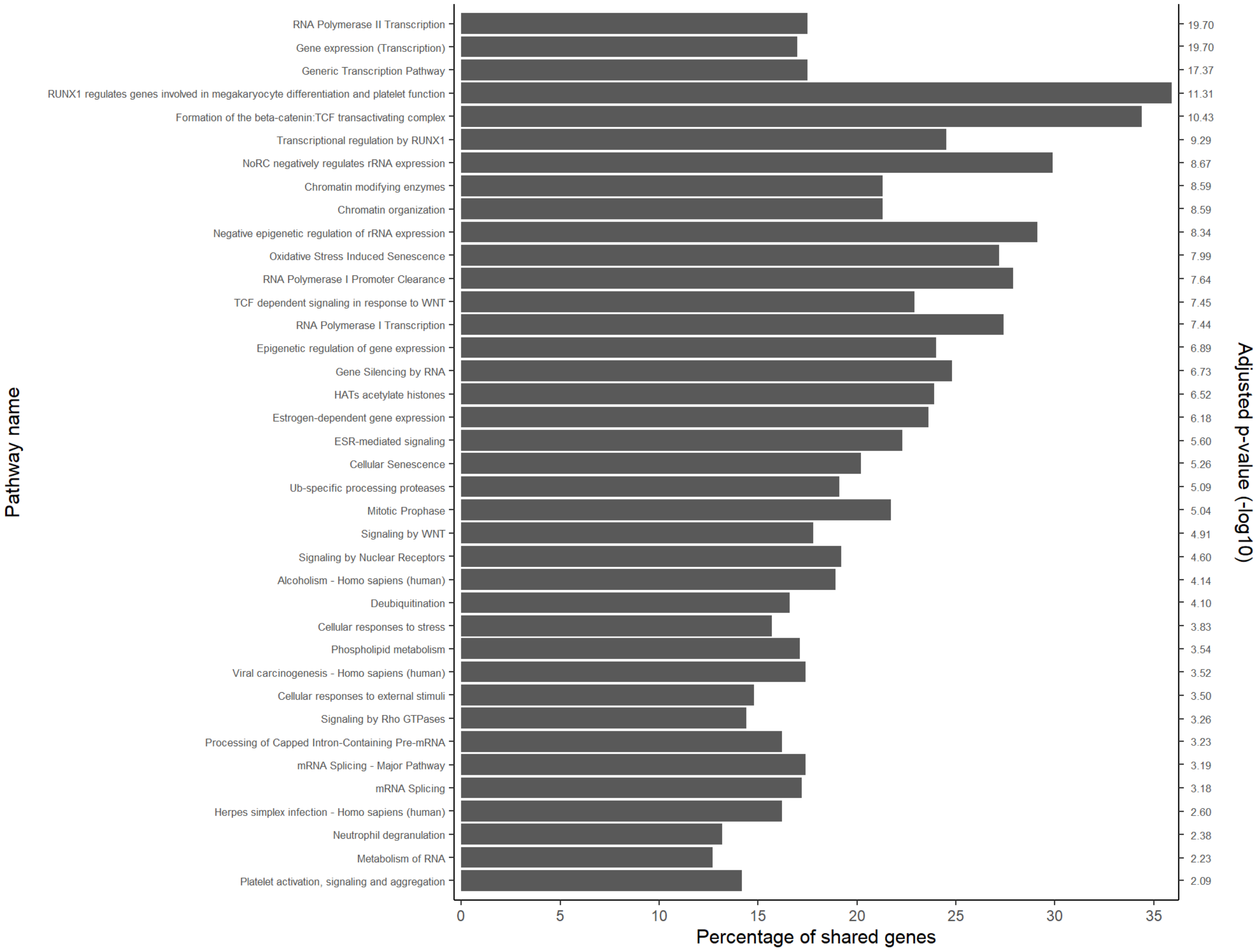


**Figure S5b:** Pathways significantly overrepresented in the DEGs between RD and CTRL. ORA made by ConsensusPathDB using DEGs (FDR <0.05, FC >0) with minimum overlap of 15 genes in pathway and p-value cut-off of 0.01. The percentage of DEGs associated with each biological process is shown along the x-axis and -log10-transformed p-values on the y-axis.

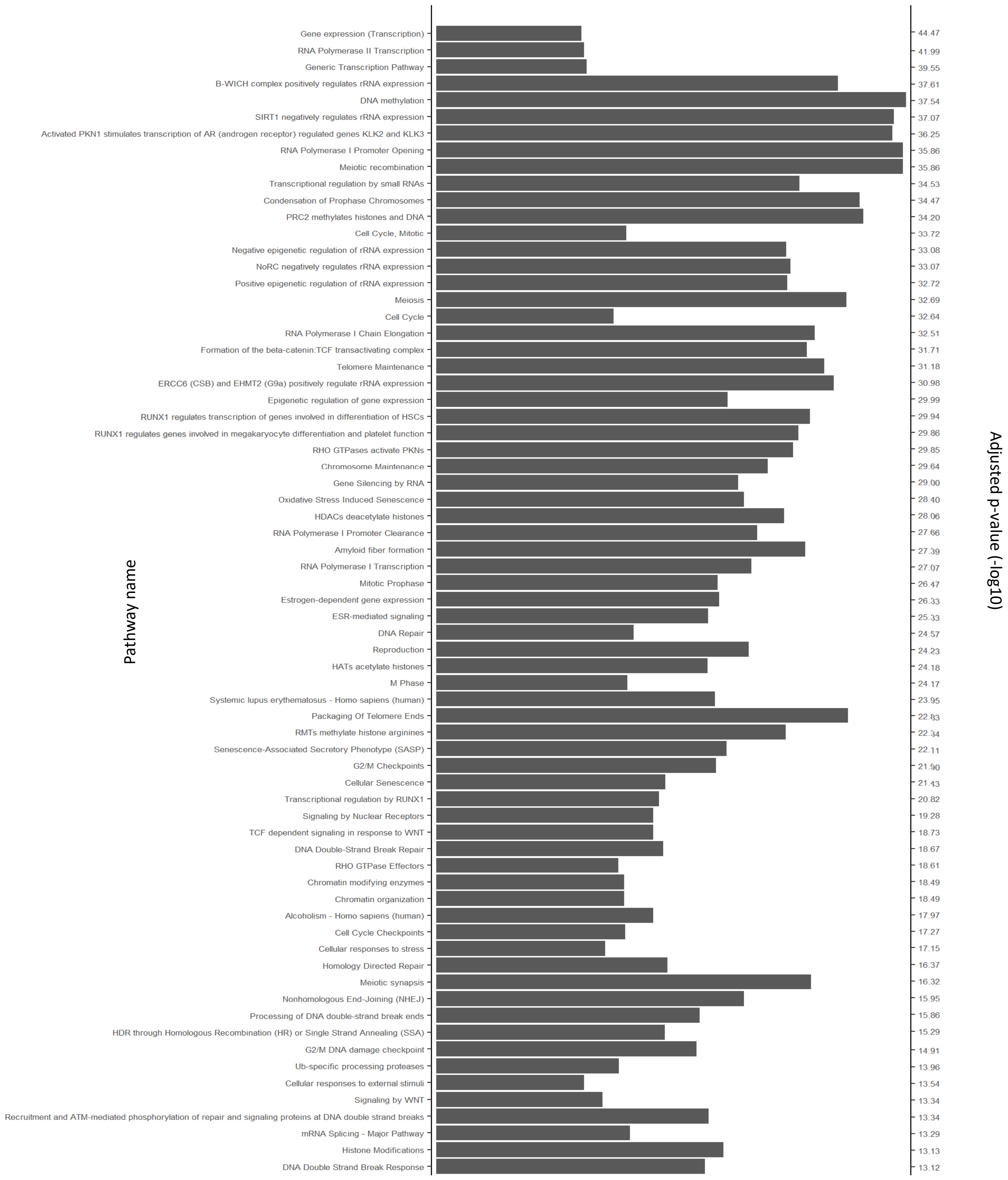
Gene expression (Transcription)
RNA Polymerase II Transcription
Generic Transcription Pathway
B-WICH complex positively regulates rRNA expression
DNA methylation
SIRT1 negatively regulates rRNA expression
Activated PKN1 stimulates transcription of AR (androgen receptor) regulated genes KLK2 and KLK3
RNA Polymerase I Promoter Opening
Meiotic recombination
Transcriptional regulation by small RNAs
Condensation of Prophase Chromosomes
PRC2 methylates histones and DNA
Cell Cycle, Mitotic
Negative epigenetic regulation of rRNA expression
NoRC negatively regulates rRNA expression
Positive epigenetic regulation of rRNA expression
Meiosis
Cell Cycle
RNA Polymerase I Chain Elongation
Formation of the beta-catenin:TCF transactivating complex
Telomere Maintenance
ERCC6 (CSB) and EHMT2 (G9a) positively regulate rRNA expression
Epigenetic regulation of gene expression
RUNX1 regulates transcription of genes involved in differentiation of HSCs
RUNX1 regulates genes involved in megakaryocyte differentiation and platelet function
RHO GTPases activate PKNs
Chromosome Maintenance
Gene Silencing by RNA
Oxidative Stress Induced Senescence
HDACs deacetylate histones
RNA Polymerase I Promoter Clearance
Amyloid fiber formation
RNA Polymerase I Transcription
Mitotic Prophase
Estrogen-dependent gene expression
ESR-mediated signaling
DNA Repair
Reproduction
HATs acetylate histones
M Phase
Systemic lupus erythematosus - Homo sapiens (human)
Packaging Of Telomere Ends
RMTs methylate histone arginines
Senescence-Associated Secretory Phenotype (SASP)
G2/M Checkpoints
Cellular Senescence
Transcriptional regulation by RUNX1
Signaling by Nuclear Receptors
TCF dependent signaling in response to WNT
DNA Double-Strand Break Repair
RHO GTPase Effectors
Chromatin modifying enzymes
Chromatin organization
Alcoholism - Homo sapiens (human)
Cell Cycle Checkpoints
Cellular responses to stress
Homology Directed Repair
Meiotic synapsis
Nonhomologous End-Joining (NHEJ)
Processing of DNA double-strand break ends
HDR through Homologous Recombination (HR) or Single Strand Annealing (SSA)
G2/M DNA damage checkpoint
Ub-specific processing proteases
Cellular responses to external stimuli
Signaling by WNT
Recruitment and ATM-mediated phosphorylation of repair and signaling proteins at DNA double strand breaks
mRNA Splicing - Major Pathway
Histone Modifications
DNA Double Strand Break Response
Pathway name
44.47
41.99
39.55
37.61
37.54
37.07
36.25
35.86
35.86
34.53
34.47
34.20
33.72
33.08
33.07
32.72
32.69
32.64
32.51
31.71
31.18
30.98
29.99
29.94
29.86
29.85
29.64
29.00
28.40
28.06
27.66
27.39
27.07
26.47
26.33
25.33
24.57
24.23
24.18
24.17
23.95
22.83
22.34
22.11
21.90
21.43
20.82
19.28
18.73
18.67
18.61
18.49
18.49
17.97
17.27
17.15
16.37
16.32
15.95
15.86
15.29
14.91
13.96
13.54
13.34
13.34
13.29
13.13
13.12
Adjusted p-value (-log10)

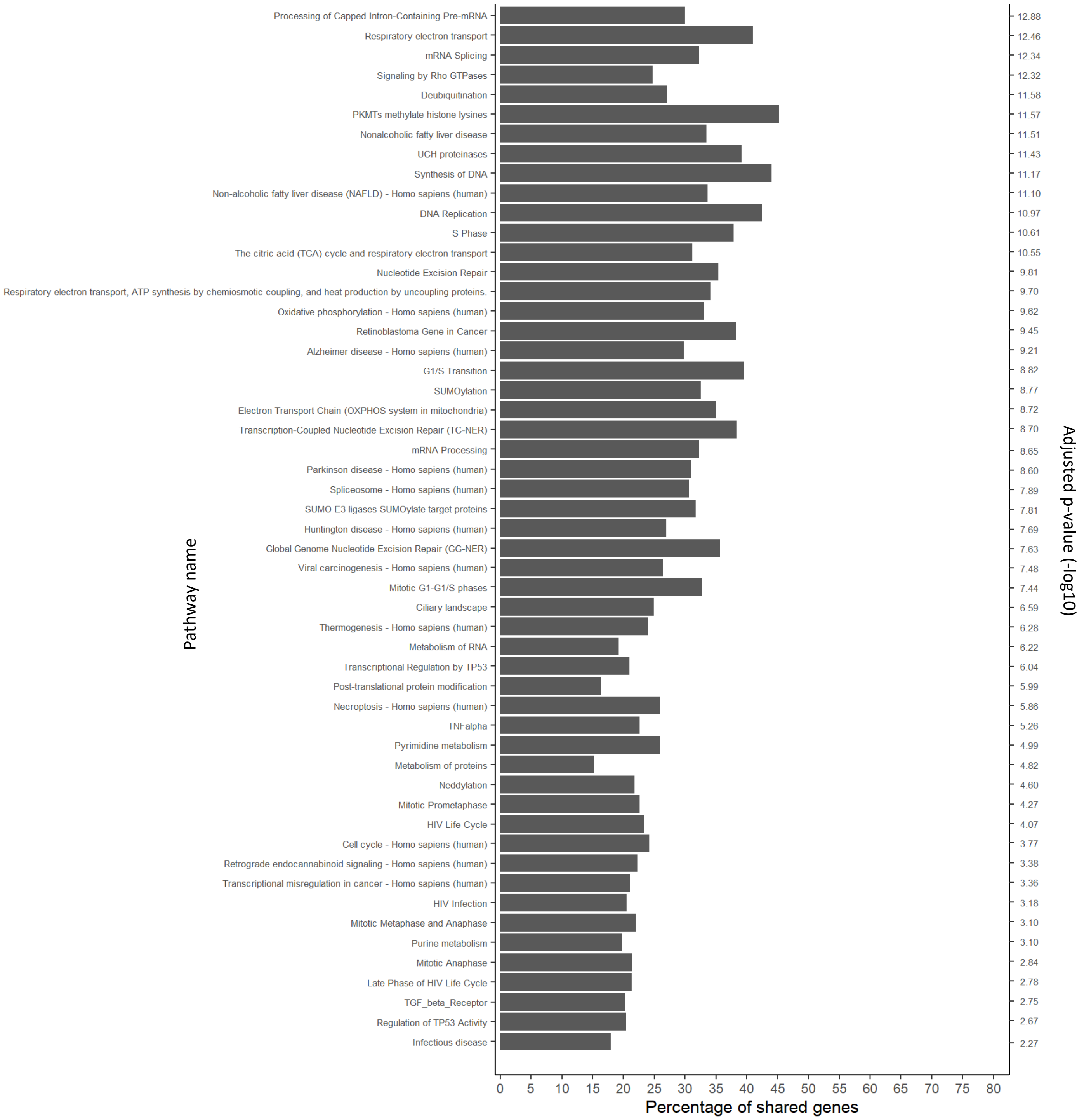


**Figure S5c:** Pathways significantly overrepresented in the DEGs between PD and CTRL. ORA made by ConsensusPathDB using DEGs (FDR <0.05, FC >0) with minimum overlap of 15 genes in pathway and p-value cut-off of 0.01. The percentage of DEGs associated with each biological process is shown along the x-axis and -log10-transformed p-values on the y-axis.

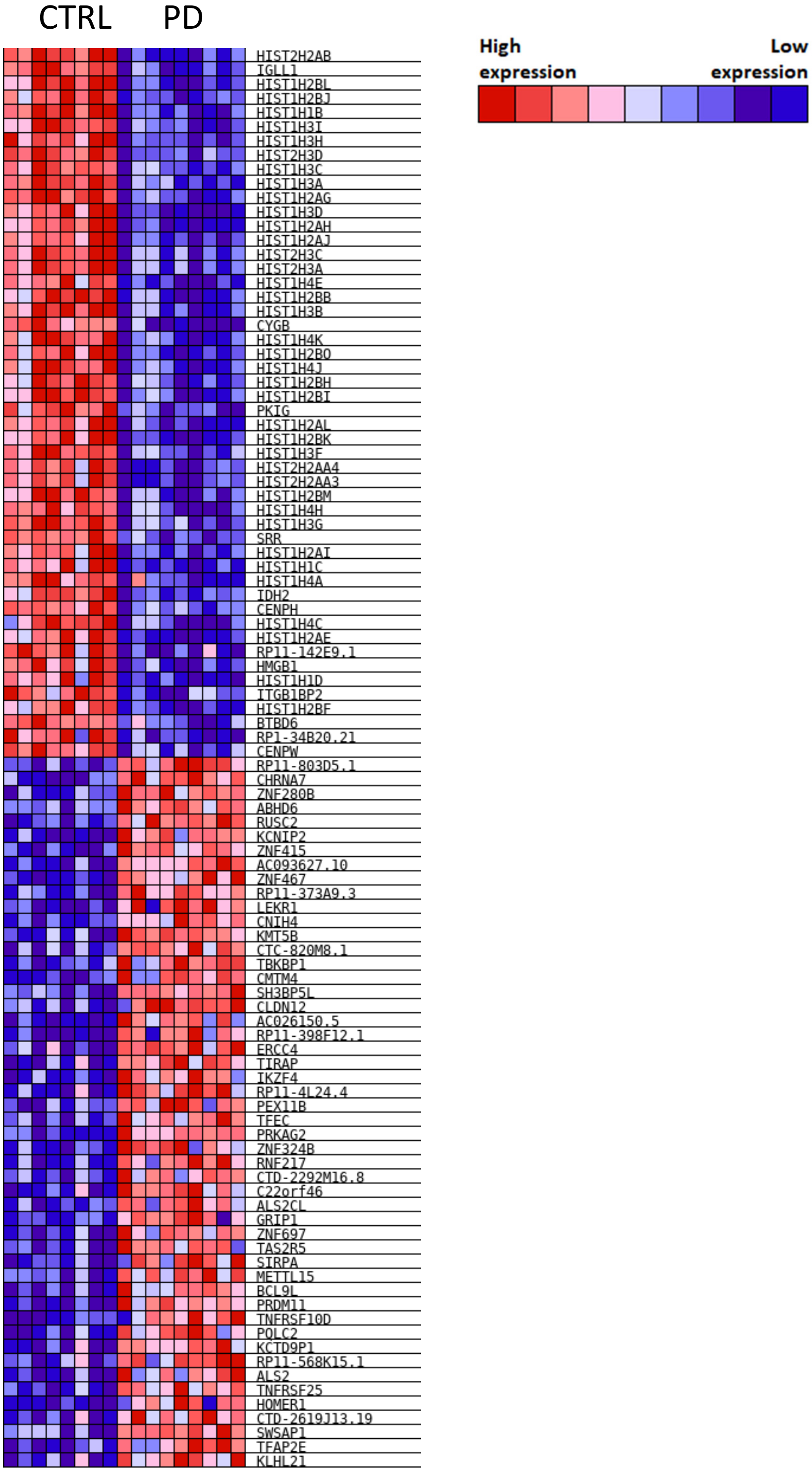


**Figure S6:** Heatmap of the top 50 up and downregulated genes in the PD vs. CTRL samples as identified by GSEA.

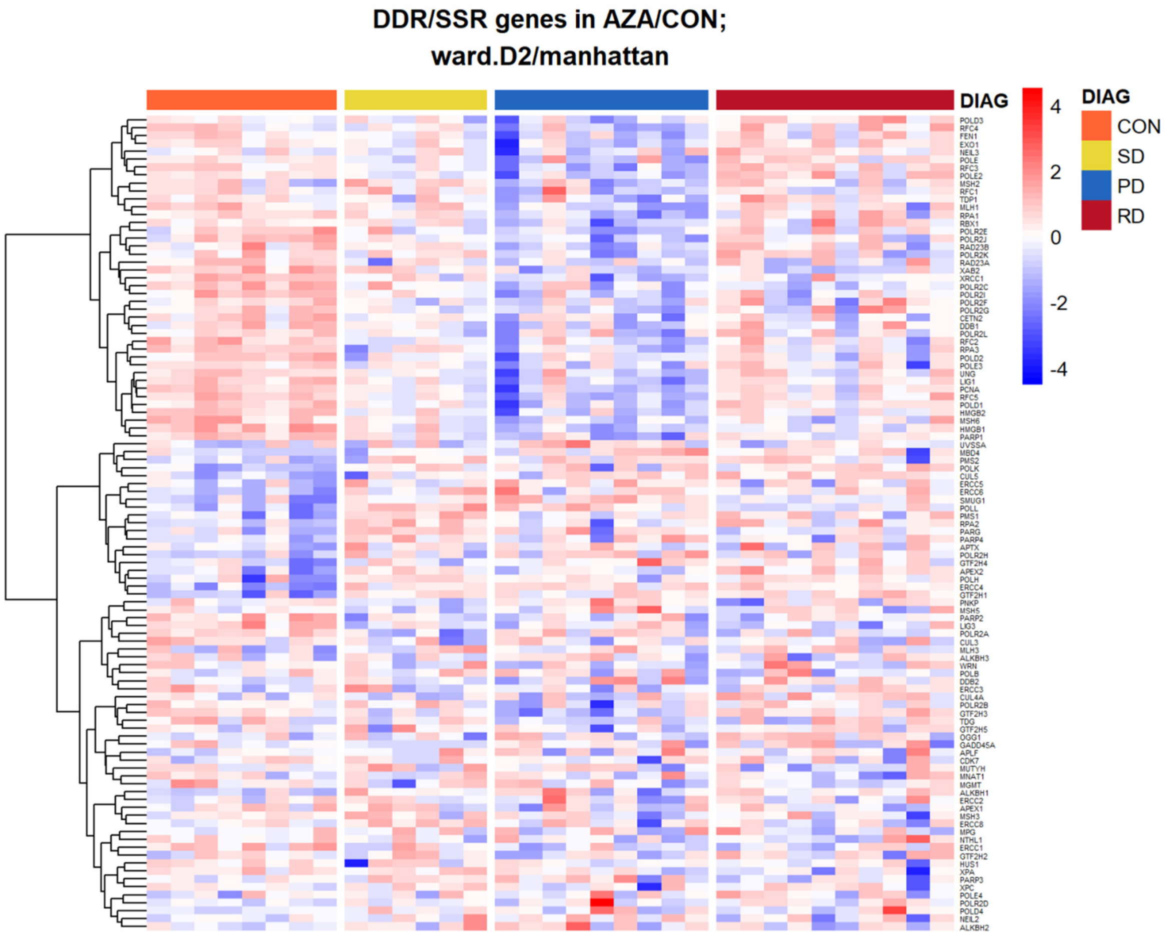


**Figure S7a:** Heatmap representation of differential expression of DNA single-strand break repair genes in the CD34+ HSPCs from AZA pre-treatment patients (RD, SD, PD) and normal controls (CON). The list of genes (n = 105) was adapted from Pearl et al (2015) [1].

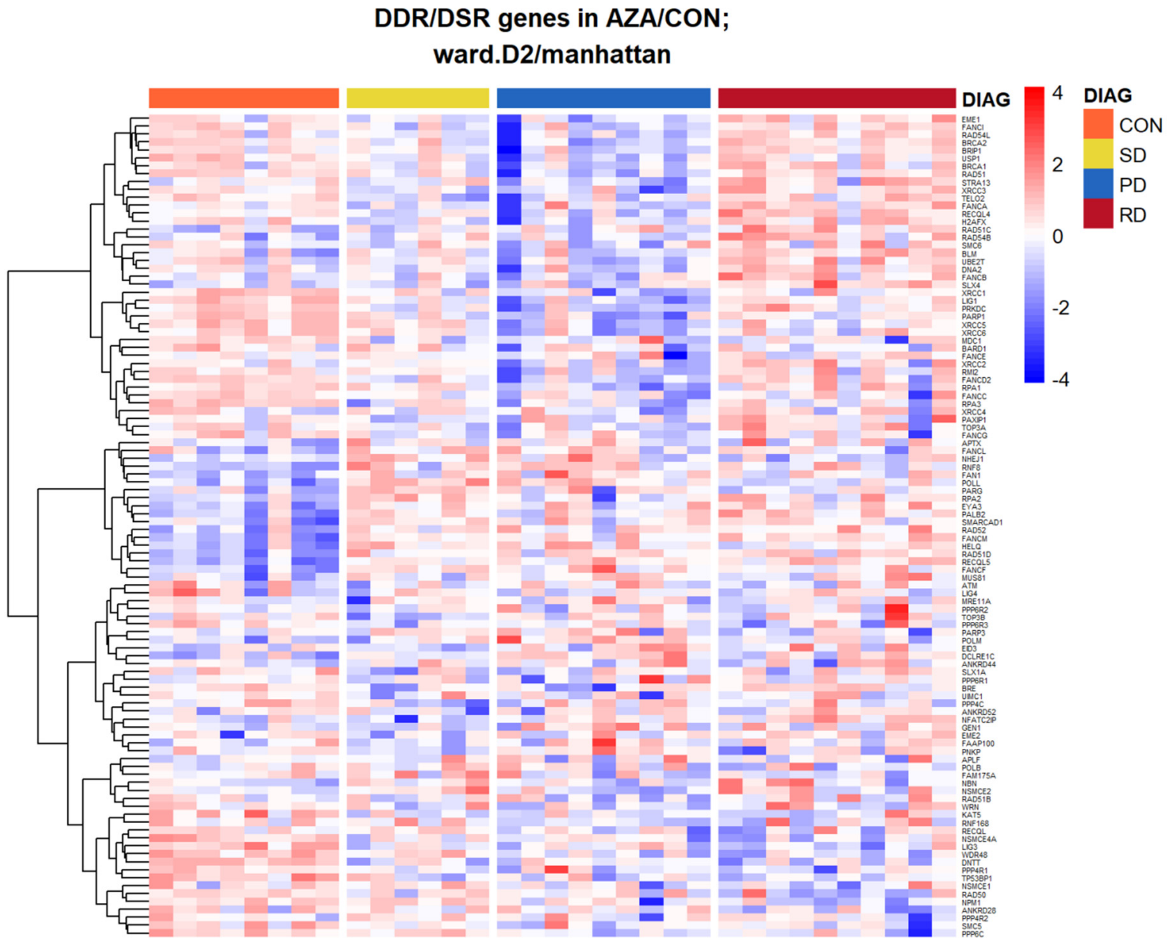


**Figure S7b:** Heatmap representation of differential expression of DNA double-strand break repair genes in the CD34+ HSPCs from AZA pre-treatment patients (RD, SD, PD) and normal controls (CON). The list of genes (n = 105) was adapted from Pearl et al (2015) [1].

**Table S5:** The top 10 up and downregulated DEGs between RD and PD. Up and downregulated genes are indicated in red and blue, respectively.

| Upregulated DEGs | | | | | |
|---|---|---|---|---|---|
| **Gene ID** | **Gene symbol** | **LogFC** | **LogCPM** | **p-value** | **FDR** |
| ENSG00000196565 | HBG2 | 4.153118783 | 4.120296678 | 0.00221902 | 0.0629817 |
| ENSG00000213934 | HBG1 | 4.148562311 | 4.135848035 | 0.002606297 | 0.06733715 |
| ENSG00000163736 | PPBP | 3.868966411 | 6.790678829 | 0.000551591 | 0.033126168 |
| ENSG00000101162 | TUBB1 | 3.852763602 | 6.11622266 | 0.000143233 | 0.016810123 |
| ENSG00000259207 | ITGB3 | 3.558529841 | 7.604599917 | 0.000216148 | 0.020562717 |
| ENSG00000284931 | CTD-2643I7.4 | 3.523549238 | 4.117788735 | 0.011383826 | 0.139089305 |
| ENSG00000119862 | LGALSL | 3.514399333 | 5.388804262 | 0.000150616 | 0.01703623 |
| ENSG00000178732 | GP5 | 3.416159009 | 3.765729738 | 0.000142354 | 0.016810123 |
| ENSG00000137801 | THBS1 | 3.414788869 | 8.336386456 | 0.00073137 | 0.037521876 |
| ENSG00000173083 | HPSE | 3.331855749 | 4.84505582 | 7.36843E-06 | 0.003649912 |
| **Downregulated DEGs** | | | | | |
| **Gene ID** | **Gene symbol** | **LogFC** | **LogCPM** | **p-value** | **FDR** |
| ENSG00000210082 | MT-RNR2 | -3.28969971 | 9.22991298 | 1.65084E-10 | 2.37144E-06 |
| ENSG00000280800 | CH507-513H4.6 | -3.282208654 | 6.156719282 | 5.36197E-07 | 0.000600408 |
| ENSG00000280614 | CH507-513H4.4 | -3.280686329 | 6.159268164 | 5.39059E-07 | 0.000600408 |
| ENSG00000281181 | CH507-513H4.3 | -3.278352132 | 6.163105589 | 5.43356E-07 | 0.000600408 |
| ENSG00000281383 | CH507-513H4.5 | -3.211821676 | 5.390666297 | 6.81518E-07 | 0.000699286 |
| ENSG00000283458 | RP11-1I2.1 | -3.210937176 | 3.057280734 | 0.011005701 | 0.136972003 |
| ENSG00000160321 | ZNF208 | -3.116835177 | 3.000686238 | 0.00460108 | 0.088746416 |
| ENSG00000275215 | RNA5-8SN4 | -3.021873157 | 6.246345948 | 4.42778E-08 | 0.000160525 |
| ENSG00000278233 | RNA5-8SN3 | -3.020919174 | 6.249364085 | 4.46989E-08 | 0.000160525 |
| ENSG00000229236 | TTTY10 | -3.004284095 | 1.51872652 | 0.137565544 | 0.449371553 |

**Table S6:** Statistical analysis of differences in the levels of selected plasma metabolites between individual clinical groups of AZA-pre-treatment patients and normal controls.

| CTRL | Citrate | cis-Aconitate | Isocitrate | α-KG | Succinate | Fumarate | Malate | L-2HG | D-2HG | 3-PG | PEP | Lactate |
|---|---|---|---|---|---|---|---|---|---|---|---|---|
| **PD** | 0.01 (*) | 0.85 | 0.97 | 0.01 (*) | 0.03 (*) | 0.04 (*) | 0.54 | 0.01 (*) | 0.37 | 0.37 | 0.86 | 0.11 |
| **RD** | 0.73 | 0.73 | 0.33 | 0.82 | 0.18 | 0.01 (*) | 0.11 | 0.02 (*) | 0.08 | 0.15 | 0.79 | 0.22 |
| **SD** | 0.09 | 1.00 | 0.45 | 0.84 | 0.32 | 0.00(**) | 0.01 (*) | 0.20 | 0.32 | 0.41 | 0.62 | 0.03 (*) |

| PD | Citrate | cis-Aconitate | Isocitrate | α-KG | Succinate | Fumarate | Malate | L-2-HG | D-2-HG | 3-PG | PEP | Lactate |
|---|---|---|---|---|---|---|---|---|---|---|---|---|
| **CTRL** | 0.01 (*) | 0.85 | 0.97 | 0.01 (*) | 0.03 (*) | 0.04 (*) | 0.54 | 0.01 (*) | 0.37 | 0.37 | 0.85 | 0.11 |
| **RD** | 0.01 (*) | 0.44 | 0.51 | 0.10 | 0.22 | 0.44 | 0.51 | 0.86 | 0.65 | 0.57 | 1.00 | 0.96 |
| **SD** | 0.27 | 0.51 | 0.25 | 0.19 | 0.25 | 0.24 | 0.08 | 0.20 | 1.00 | 0.95 | 0.63 | 0.20 |

| RD | Citrate | cis-Aconitate | Isocitrate | α-KG | Succinate | Fumarate | Malate | L-2-HG | D-2-HG | 3-PG | PEP | Lactate |
|---|---|---|---|---|---|---|---|---|---|---|---|---|
| **CTRL** | 0.73 | 0.73 | 0.33 | 0.82 | 0.18 | 0.01 (*) | 0.11 | 0.02 (*) | 0.08 | 0.15 | 0.79 | 0.22 |
| **PD** | 0.01 (*) | 0.44 | 0.51 | 0.10 | 0.22 | 0.44 | 0.51 | 0.86 | 0.65 | 0.57 | 1.00 | 0.96 |
| **SD** | 0.17 | 0.83 | 0.98 | 0.95 | 0.98 | 0.78 | 0.27 | 0.30 | 0.51 | 0.40 | 0.63 | 0.25 |

| SD | Citrate | cis-Aconitate | Isocitrate | α-KG | Succinate | Fumarate | Malate | L-2-HG | D-2-HG | 3-PG | PEP | Lactate |
|---|---|---|---|---|---|---|---|---|---|---|---|---|
| **CTRL** | 0.09 | 1.00 | 0.45 | 0.84 | 0.32 | 0.00(**) | 0.01 (*) | 0.20 | 0.32 | 0.41 | 0.62 | 0.03 (*) |
| **RD** | 0.17 | 0.83 | 0.98 | 0.95 | 0.98 | 0.78 | 0.27 | 0.30 | 0.51 | 0.40 | 0.63 | 0.25 |
| **PD** | 0.27 | 0.51 | 0.25 | 0.19 | 0.25 | 0.24 | 0.08 | 0.20 | 1.00 | 0.95 | 0.63 | 0.20 |

The Mann Whitney U test, $p < 0.05$ (*), $p < 0.001$ (**). The light –dark blue color scale indicates decreased metabolite level and the light – dark orange color scale shows increased metabolite level. Patients with *IDH2* mutations were excluded from the analyses. CTRL – controls, PD – progressive disease, RD – responders, SD – stable disease, α-KG – alpha-ketoglutarate, L- 2HG – L-2-hydroxyglutarate, D-2HG - D-2-hydroxyglutarate, 3-PG – 3-phosphoglycerate, PEP –phosphoenolpyruvate.

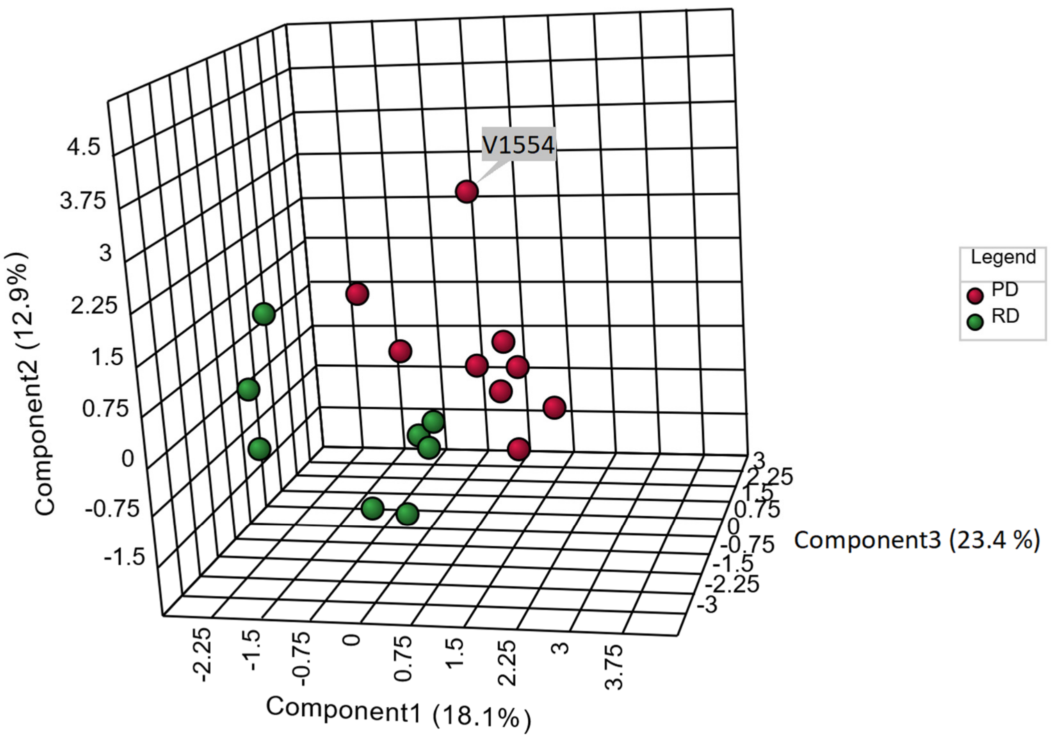


**Figure S8a:** Principal component analysis (PCA) of plasma metabolic profile of the AZA-pre-treatment PD and RD patients. A Patient with *IDH2* mutation is depicted by V1554 symbol. The samples were distinguished by a 19-metabolites signature (see **Supplementary Materials and Methods**).

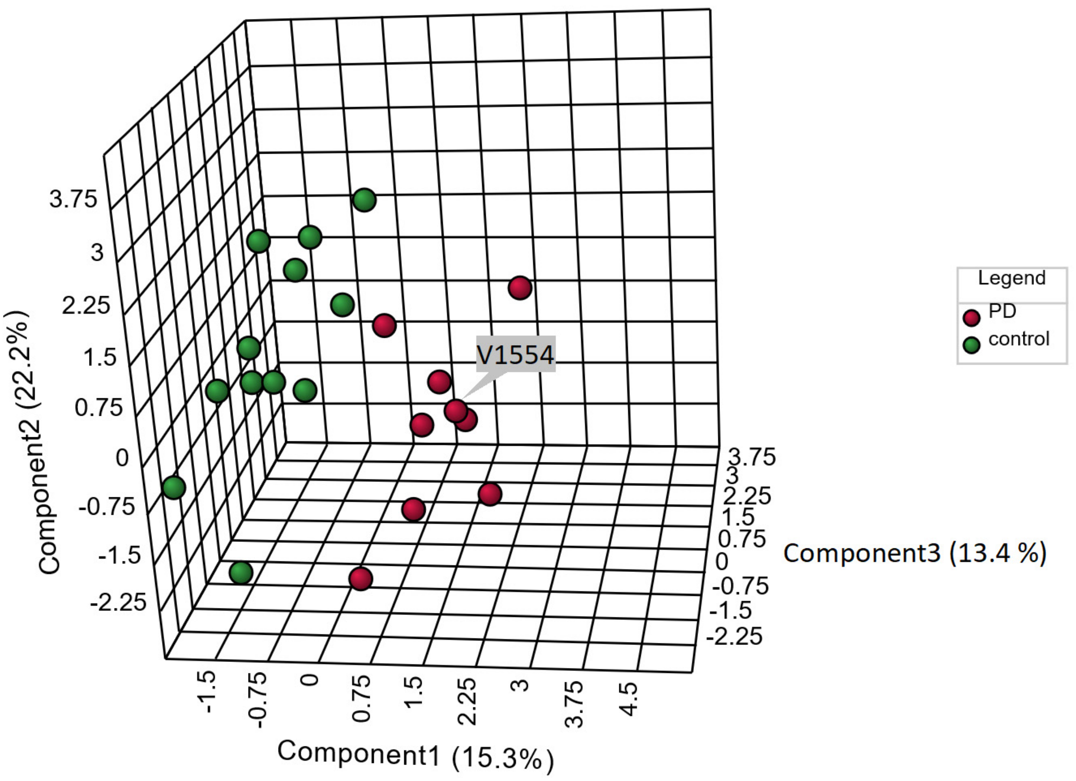


**Figure S8b:** PCA of plasma metabolic profile of the AZA-pre-treatment PD patients and normal controls. A Patient with *IDH2* mutation is depicted by V1554 symbol. The samples were distinguished by a 19-metabolites signature (see **Supplementary Materials and Methods**). PCA-3D plot displays a distinct profile between PD and CRTL samples.

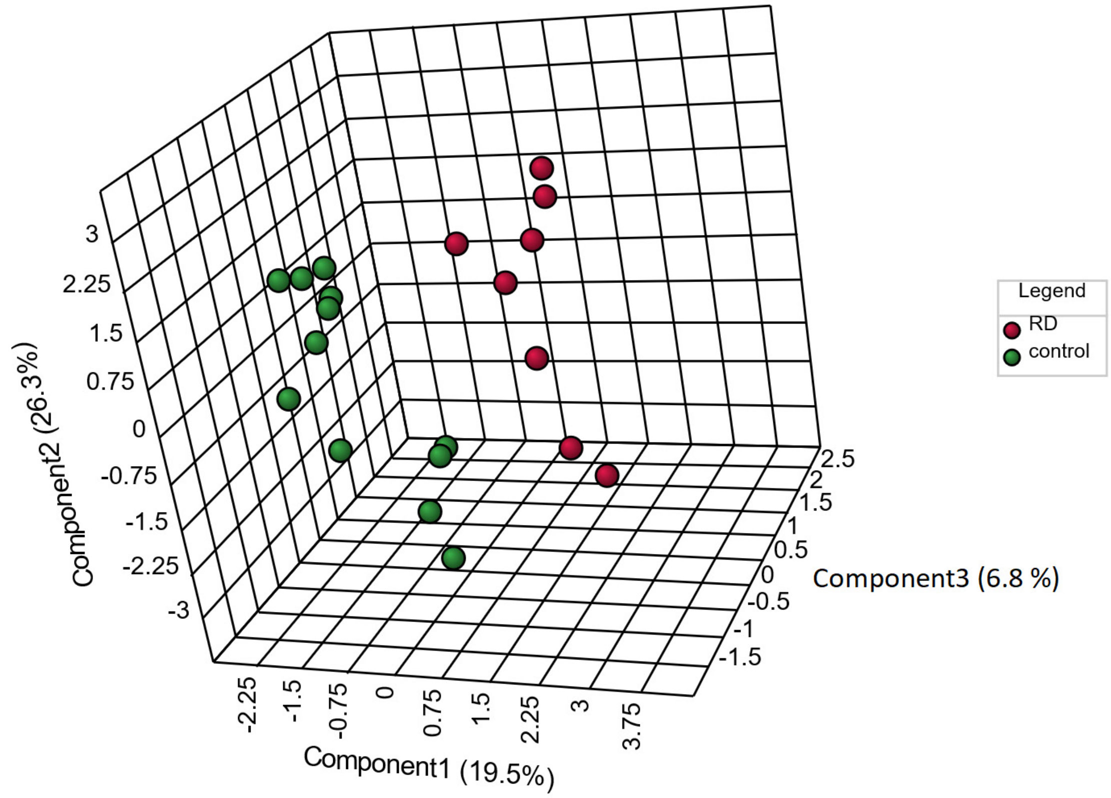


**Figure S8c:** PCA of plasma metabolic profile of the AZA-pre-treatment RD cohort and normal controls. The samples were distinguished by a 19-metabolites signature (see **Supplementary Materials and Methods**). PCA-3D plot displays a distinct profile between RD and CRTL samples.

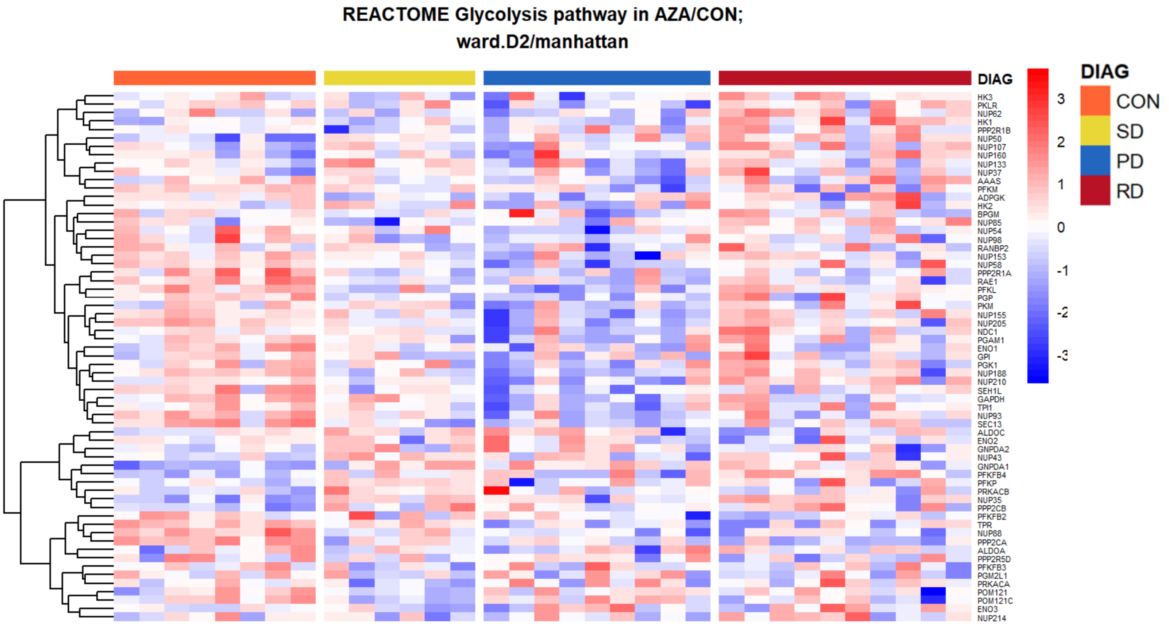


**Figure S9:** Heatmap visualization of gene expression profiles for glycolysis pathway genes (n = 63, Reactome) in the CD34+ HSPCs from AZA pre-treatment patients (RD, SD, PD) and normal controls (CON).

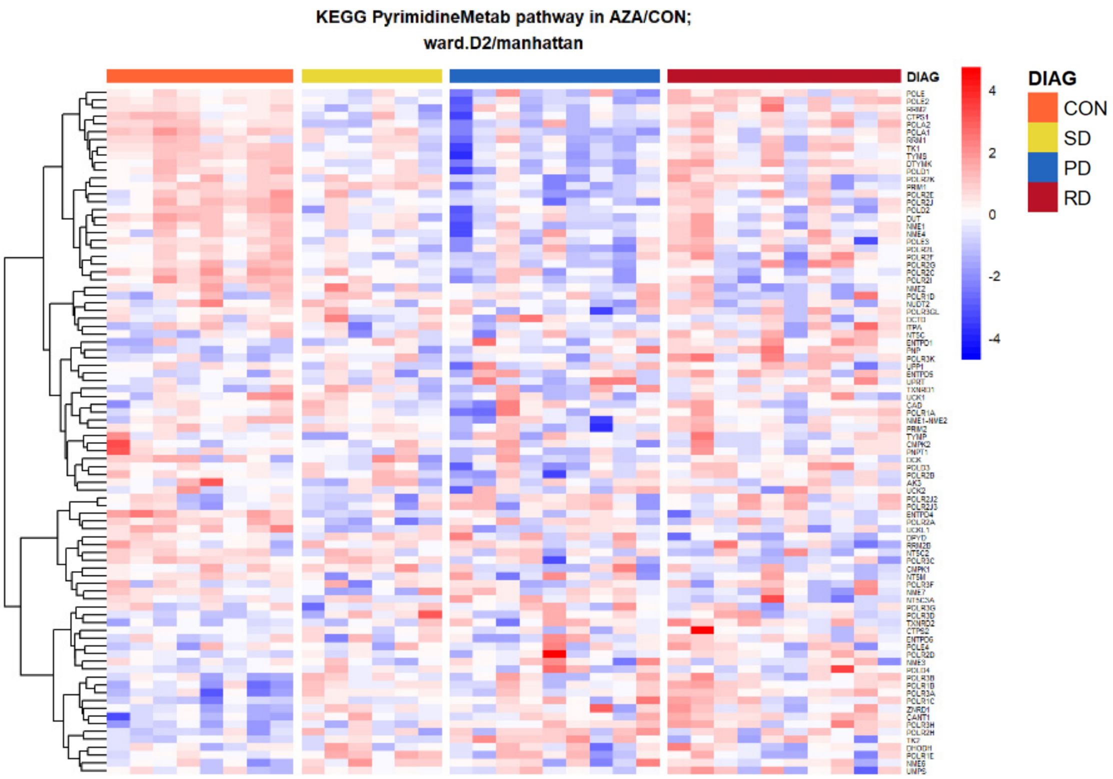


**Figure S10:** Heatmap visualization of gene expression profiles for pyrimidine metabolism genes (n = 88, KEGG) in the CD34$^{+}$ HSPCs from AZA pre-treatment patients (RD, SD, PD) and normal controls (CON).

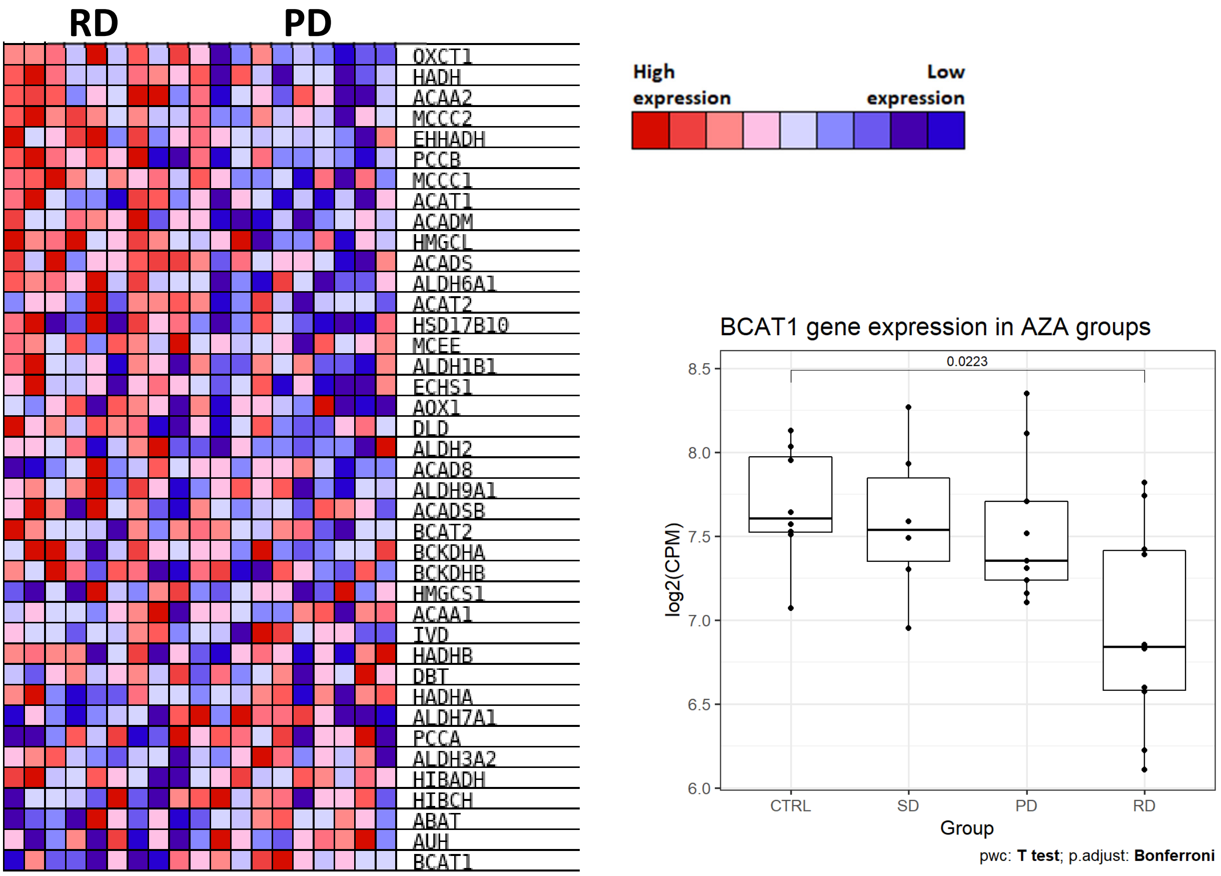


**Figure S11:** Left: Heatmap visualization of DEGs involved in valine/leucine/isoleucine degradation (n = 40, KEGG) in the CD34+ HSPCs from AZA pre-treatment RD and PD patients. Right**:** The boxplot graph of relative log2 expression of *BCAT1* in the individual clinical groups (SD, PD, RD) of AZA-pre-treatment patients (n = 24) and normal controls (n = 8). Student's t-test with Bonferroni correction, p-value shown in the graph.

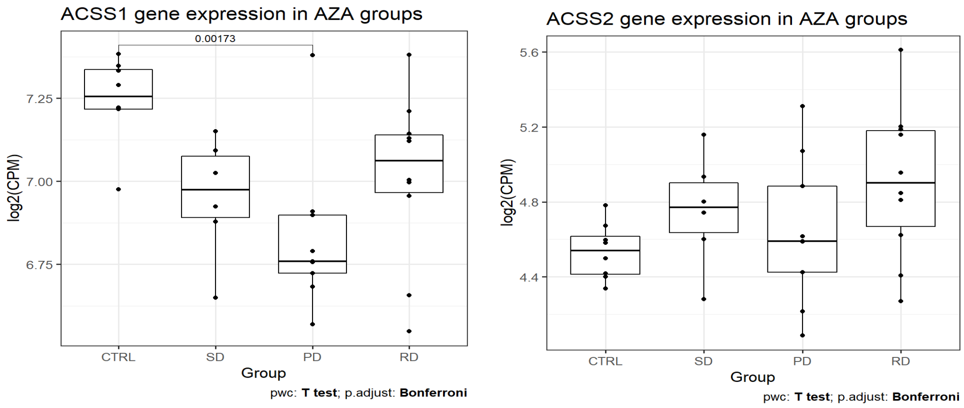


**Figure S12:** The boxplot graphs of relative log2 expression of *ACSS1* and *ACSS2* genes in the AZA-pre-treatment HSPCs from patients (n=24) and normal controls (n=8), Student's t-test with Bonferroni correction, p-value shown in the graphs.

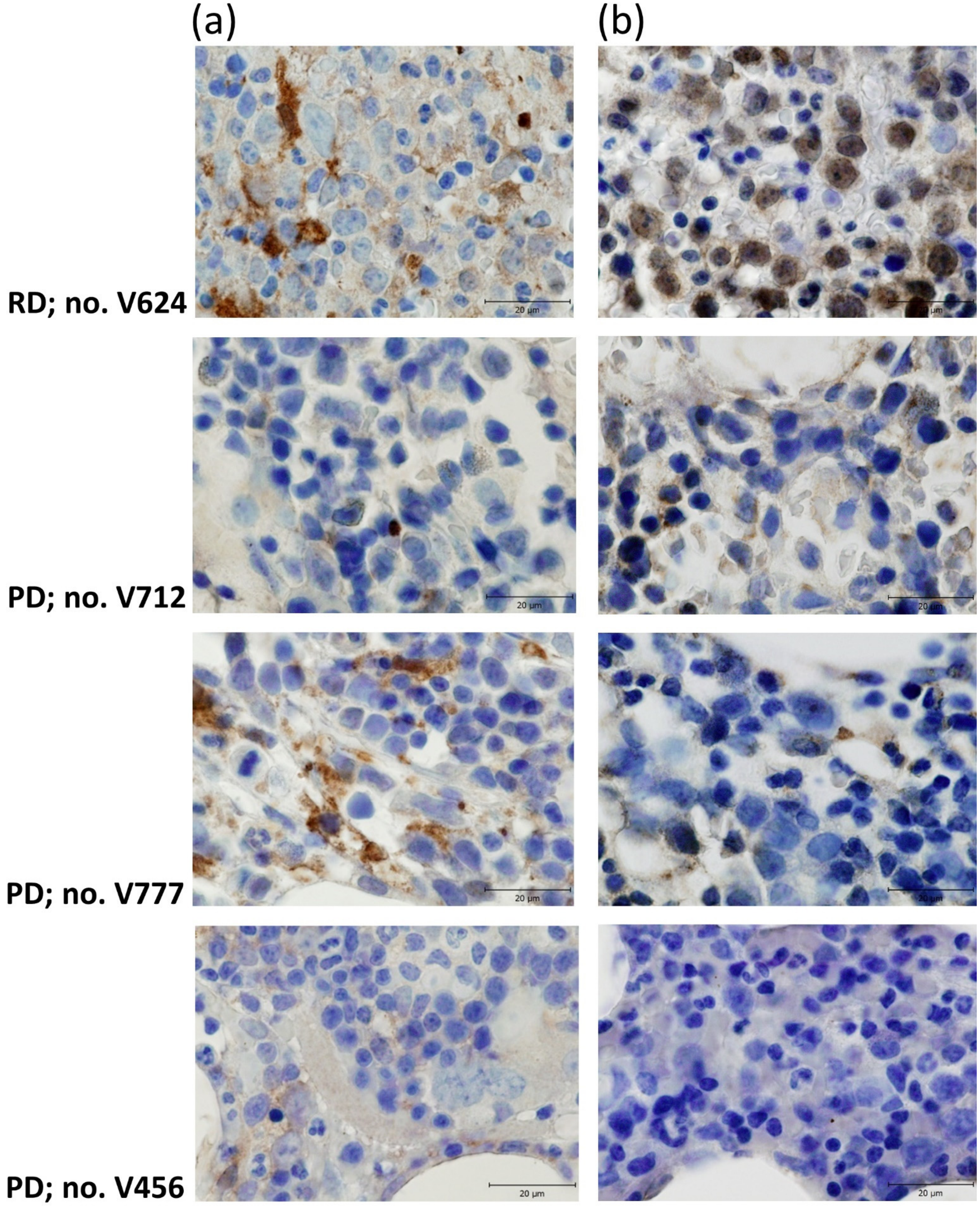


**Figure S13:** Immunohistochemistry (IHC) staining for IDH1 (a) and pan-acetyl lysine (b) in bone marrow trephine biopsies of MDS/AML-MRC patients. (a) Upper panel: Cytoplasmic staining for IDH1 in RD patient no. V624. Middle and lower panels: Weak/Intermediate cytoplasmic staining for IDH1 in three PD patients no. V712, V777, V456. (b) Nuclear pan-acetyl lysine staining in BM trephine biopsies from RD vs. PD patients. Upper panel: Strong nuclear staining documenting high level of lysine acetylation in RD patient no. V624. Middle and lower panels: Low/undetectable level of lysine acetylation in nuclei of three PD patients no. V712, V777, V456. See Supplementary Table S1 for patients' details. Scale bars, 20 µm.

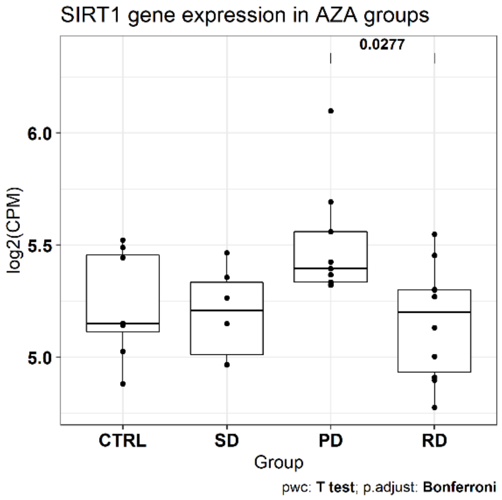


**Figure S14:** The boxplot graph of relative log2 expression for *SIRT1* gene in the AZA-pre-treatment HSPCs from patients (n=24) and normal controls (n=8). Student's t-test with Bonferroni correction, p-values shown in the graph.

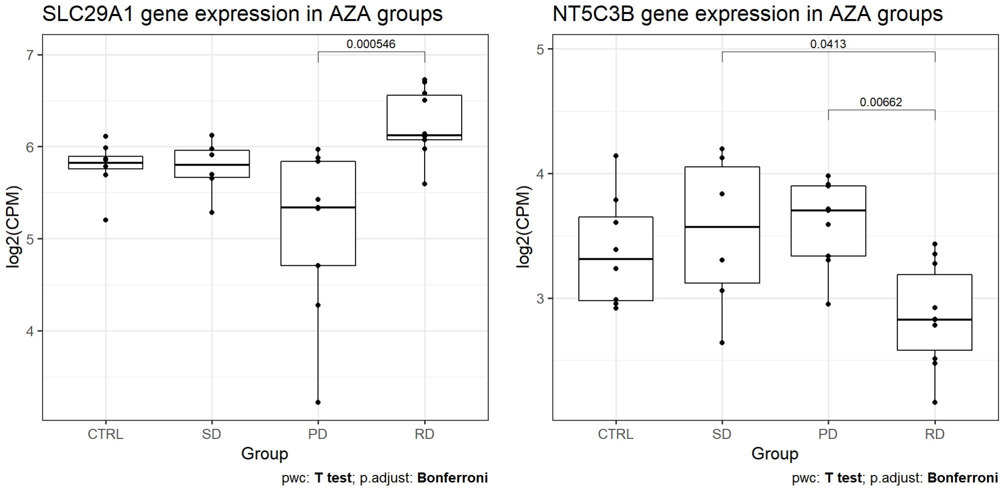


**Figure S15:** The boxplot graphs of relative log2 expression for *SLC29A1* and *NT5C3B* genes in the AZA-pre-treatment HSPCs from patients (n=24) and normal controls (n=8). Student's t-test with Bonferroni correction, p-values shown in the graphs.

**Table S7:** Distribution of myeloid and B-lymphoid subsets of $CD34^+$ cells in BM samples from 16 patients of discovery cohort, based on flow cytometry analysis. See Table S1 for patients' numbering and details.

| **Patient ID** | % of **myeloid blasts** (CD45dim, $CD13^+$, $CD33^+$, $CD117^+$, HLA-$DR^+$) from total $CD34^+$ cells | % of **B-lymphoid precursors** (CD45dim, $CD10^+$, $CD19^+$, $CD20^-$) from total $CD34^+$ cells |
|---|---|---|
| V716 | 8 | 0 |
| V1874 | 39 | 0 |
| V1441 | 8.7 | 0 |
| V1337 | 2.36 | 0.25 |
| V1394 | 6 | 0 |
| V1592 | 6.5 | 0 |
| V714 | 17 | 0 |
| V712 | 25 | 0 |
| V1297 | 10 | 2.5 |
| V1554 | 33 | 0 |
| V344 | 18 | 1.8 |
| V1788 | 29 | 0 |
| V1456 | 2 | 1 |
| V1884 | 12 | 0 |
| V655 | 15 | 1.1 |
| V1279 | 8 | 0 |

## References:


1. Pearl, L.H.; Schierz, A.C.; Ward, S.E.; Al-Lazikani, B.; Pearl, F.M.G. Therapeutic Opportunities within the DNA Damage Response. *Nature Reviews Cancer* **2015**, *15*, 166–180, doi:10.1038/nrc3891.
